# Emerging engineering strategies in bone organoids: From biomimetic scaffolds to dynamic microenvironmental stimulation

**DOI:** 10.1016/j.bioactmat.2026.04.008

**Published:** 2026-04-17

**Authors:** Jiamian Han, Hongcheng Gu, Zhongze Gu

**Affiliations:** State Key Laboratory of Digital Medical Engineering, School of Biological Science and Medical Engineering Southeast University, Nanjing, 210096, China

**Keywords:** Bone organoids, Organ-on-a-chip, Stimuli-responsive materials, Biomimetic scaffolds, Mechanotransduction

## Abstract

Bone organoids have emerged as transformative models for studying bone development and disease by recapitulating complex cellular ecosystems *in vitro*. However, unlike soft tissues, the unique mineralized matrix and highly dynamic mechanical environment of bone pose significant challenges to classical self-assembly strategies. Consequently, engineering strategies are not merely auxiliary but essential for constructing functional bone organoids. This review provides a comprehensive overview of advanced engineering strategies designed to overcome these biological hurdles. We critically examine the integration of dynamic mechanical microenvironments and the design of biomimetic topologies for guiding cell fate. Furthermore, we explore the application of bone organoids in disease modeling while addressing inherent limitations. Potential solutions based on the convergence of 3D bioprinting, microfluidic organ-on-a-chip systems, and artificial intelligence are proposed. We anticipate that deep interdisciplinary collaboration will accelerate the transition of bone organoids from theoretical exploration to clinical personalized medicine, bridging the gap between basic research and regenerative therapies.

## Highlights

• Engineering strategies are defined as essential for structural bone organoids.
• Biomimetic scaffolds with TPMS and hierarchical topology guide cell fate.
• Stimuli-responsive materials enable dynamic mechanical microenvironments.
• Integrated bone-on-a-chip platforms facilitate vascularization and disease modeling.

## Graphical abstract

**Figure:**
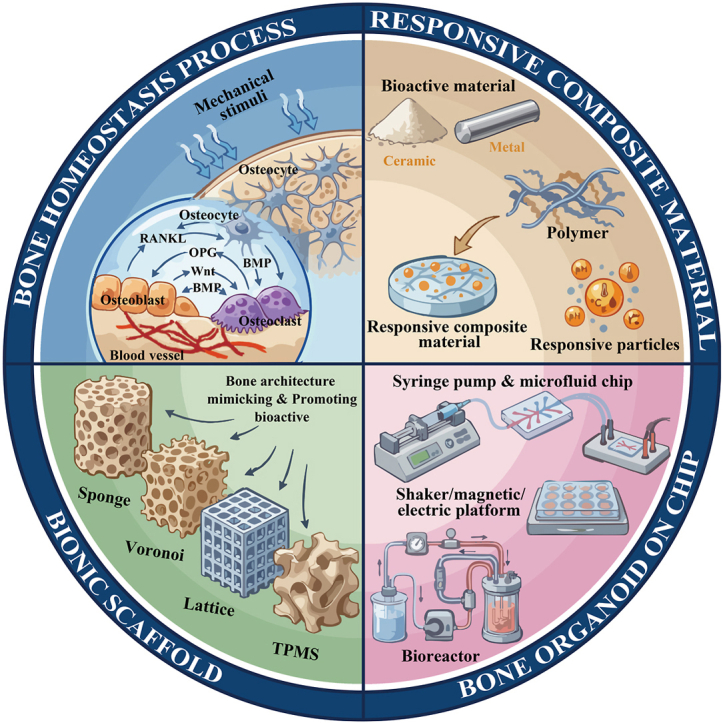

## 1 Introduction

Bone diseases, including osteosarcoma, osteoporosis (OP), osteoarthritis (OA) and osteomyelitis (OM), have become a major public health problem affecting the health of hundreds of millions of people around the world [1]. These diseases not only lead to pain, dysfunction and a significant decline in the quality of life of patients, but also bring heavy economic burden to the social medical system due to their high incidence, long treatment cycle and high recurrence rate [2]. Although significant advances have been made in two-dimensional (2D) cell culture and animal models, the inherently complex microenvironment and diverse cellular composition of natural bone make these two model systems fundamentally limited in their physiological relevance and predictive ability for clinical translation [3], [4].

As a breakthrough in the field of regenerative medicine, organoid technology provides unprecedented opportunities for disease research. Generally, organoids are three-dimensional micro-organs formed by pluripotent stem cells or tissue-specific progenitor cells through the self-assembly process, which can highly simulate the cell composition, spatial structure and physiological function of the source organ *in vitro* [5]. However, for bone, a highly sclerotic, load-bearing organ lacking epithelial tissue, complete self-assembly often results in mineralized nodules lacking complex structures, which cannot reproduce haversian system or trabecular structure of native bone. Therefore, the bone organoids discussed in this review refer specifically to engineered bone organoids. That is, the biomaterial scaffold is used to provide the necessary initial physical template and mechanical support to guide the orderly assembly and functional differentiation of cells *in vitro*, so as to provide an ideal model for the study of bone development, homeostasis maintenance and disease occurrence [[6], [7], [8]]. More importantly, bone organoids have great potential in personalized medicine, and patients’ mesenchymal stem cells (MSCs) or specific induced pluripotent stem cells (iPSCs) can be used to construct disease models for drug sensitivity testing and treatment regimen optimization [9]. In terms of high-throughput drug screening, bone organoid technology can accelerate the development of new therapies [10], [11].

Despite the technical promise of bone organoids, the field of research is still in its infancy and there are many challenges to be addressed [12]. Unlike bone tissue engineering (BTE), the primary goal of a bone organoid is to recapitulate the autonomous developmental trajectories of the skeleton, rather than using scaffolds providing immediate, rigid mechanical support to repair critical-sized defects. Therefore, the biomaterial scaffold used to create bone organoid should shift from acting as static structural fillers to morphogenetic templates that guide and confine cellular self-organization. Besides, a static template alone is insufficient to capture the complexity of skeletal development, which is inherently driven by dynamic physical forces. To translate this conceptual shift into functional *in vitro* models, the field must overcome several multi-dimensional engineering hurdles. The first is the construction of biomimetic microenvironment. How to construct 3D culture scaffolds with similar physical and chemical properties to natural bone is the basis for constructing bone organoids [[13], [14], [15]]. Secondly, bone is a highly dynamic tissue that continuously undergoes mechanical force loading, metabolic changes, and changes in signaling molecules [16]. Most of the existing organoid culture systems are in a static environment. How to establish a bioreactor system that can simulate the dynamic characteristics of bone *in vivo* (mechanical stimulation, bioelectrical stimulation, metabolic microenvironment, etc.) is the key to the functional maturation of bone organoids. Finally, vascularization not only helps to maintain the structural and functional integrity of bone-like tissues, but also supports the long-term survival and performance of organoids [17]. However, the lack of functional vascular network is the biggest limiting factor of current bone organoid technology [9], [18].

**Fig. 1:**
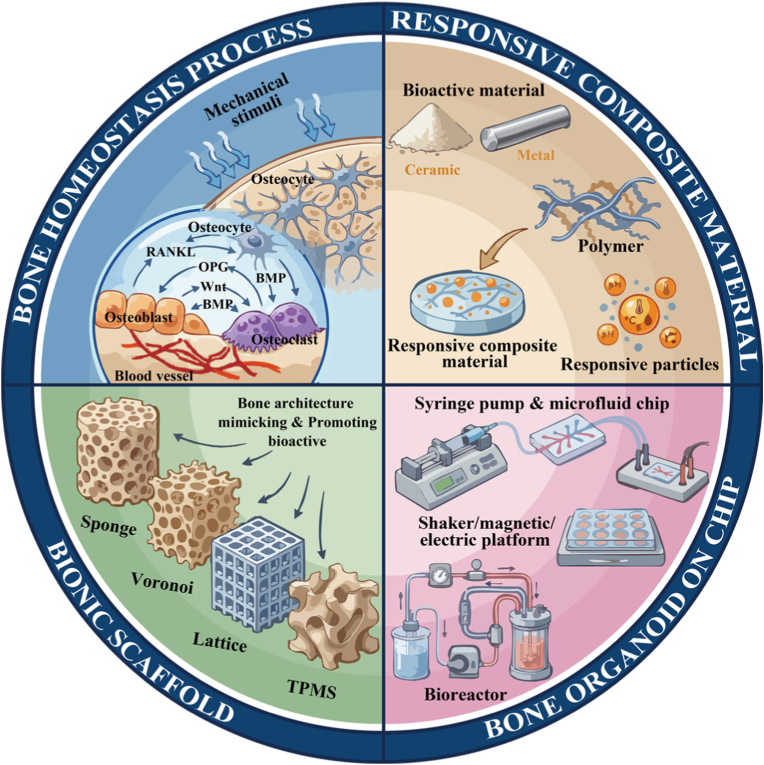
Bone metabolic processes and the engineering strategies in the construction of bone organoid, including material engineering strategies, structural engineering strategies, and organ-on-a-chip engineering strategies.

**Fig. 2:**
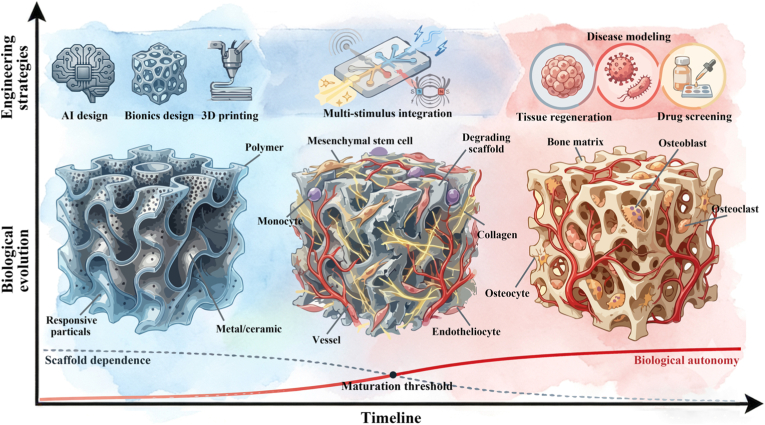
Ideal evolutionary process of engineering bone organoids.

To fundamentally distinguish bone organoids from conventional BTE, it is essential to redefine the role of engineered scaffolds. Rather than serving as permanent structural fillers, these scaffolds must function as transient morphogenetic templates. Crucially, true organoid maturation should manifest as a systematic reduction in dependence on external artificial support. As the organoid undergoes autonomous self-organization, endogenous extracellular matrix (ECM) deposition, and functional vascularization, the initial engineered template should progressively yield its mechanical and biological roles to the newly formed native tissue (See Fig. 2). To elucidate this dynamic developmental paradigm, this review provides a comprehensive overview of advanced engineering strategies for constructing functional bone organoids. We systematically examine the design of morphogenetic templates, focusing on biomimetic topologies that optimize early cellular assembly and transfer of oxygen and nutrients. We then explore the construction of dynamic microenvironments, highlighting how stimuli-responsive smart materials and external physical forces act as active regulators to drive mechanotransduction and structural evolution. Furthermore, we discuss the integration of these approaches into advanced organ-on-a-chip platforms and their subsequent applications in complex disease modeling and regenerative medicine. Finally, we propose that the deep convergence of multidisciplinary technologies including 3D bioprinting, microfluidics, and artificial intelligence will be critical for advancing the clinical translation of next-generation bone organoids (See Fig. 1).

## 2 Biological principles of engineered bone organoids

The complex three-dimensional structure of bone tissue, dynamic vascular network and the continuous remodeling ability of ECM together constitute the basis of the unique mechanical properties and physiological functions of bone [13], [15]. Bone development and regeneration are strictly driven by the physical microenvironment, which mainly follows two distinct mechanisms: intramembranous osteogenesis and endochondral osteogenesis [19]. Intramembranous osteogenesis (IO) refers to the direct differentiation of MSCs into osteoblasts and the deposition of minerals on the collagen matrix, which mainly occurs in non-load-bearing flat bones such as the skull and accounts for a small proportion of skeletal development [20]. However, the development of long bones, whose main function is load-bearing, mainly relies on endochondral ossification (EO), that is, cartilage template is formed first, followed by vascular invasion, cartilage matrix degradation and gradual replacement by bone tissue [21]. Therefore, Understanding the internal logic of bone development, the limits of material transport in a dense matrix, and the communication networks between multicellular populations are the theoretical cornerstones for designing engineering strategies. In this chapter, we will analyze these three dimensions and demonstrate why engineering is necessary to construct functional bone organoids.

### 2.1 Engineering strategies for reproducing IO

To reproduce the IO process, morphogenetic templates need to meet two requirements: guiding the high-density aggregation of MSCs and promoting the direct osteogenic differentiation of MSCs through high stiffness. Studies have shown that matrix stiffness provides a key physical cue to determine the direction of cell differentiation: hard matrices (mimicking bone ECM, 25-40 kPa) can significantly promote MSCs to differentiate into an osteogenic phenotype [22], while softer matrices (mimicking brain or cartilage tissue, 0.1-1 kPa) induce neuron-like or cartilaginous phenotypes [23], [24]. This stiffness-induced differentiation effect is closely related to the cell-intrinsic actomyosin tension system. When nonmyosin II inhibitors such as blebbistatin were used to inhibit actomyosin contractility, differences in differentiation induced by substrates of different stiffness were significantly abolished, indicating that mechanical signals are conducted through cytoskeletal tension and regulate cell fate decisions [25]. From the perspective of molecular mechanism, integrin-mediated cell-matrix adhesion plays a key role in this process. Appropriate integrin expression can activate downstream signaling pathways (such as RhoA/ROCK), regulate intracellular tension generated by actin-myosin, and then drive osteogenesis-specific gene expression by affecting cell morphology [26], [27]. In the 3D culture environment, the regulation of matrix stiffness becomes more important and complex. By designing extracellular matrices with different stiffness levels, researchers are able to study the differentiation behavior of MSCs in a 3D microenvironment that more closely resembles physiological conditions [28]. 3D stiffness microenvironment not only affects the direction of differentiation, but also regulates the spatial arrangement and intercellular communication of cells, which provides an idea for the construction of bone organoids.

### 2.2 Engineering strategies for reproducing EO

Reproducing the EO process is much more complex than reproducing the IO process because the area where EO occurs within the body is exposed to more external mechanical stimuli [[29], [30], [31]]. To faithfully recapitulate the developmental trajectory of EO, the engineered mechanical niche must dynamically evolve to drive the phase transitions from a cartilaginous template to a mineralized bone organoid. Early developmental stages are characterized by compressive cues. Dynamic compressive stimulation translates macroscopic boundary conditions into intracellular signals, primarily depending on the Integrin-FAK mechanotransduction axis [32]. As cells sense matrix deformation, the aggregation of integrin clusters activates FAK, initiating downstream biochemical cascades that govern initial self-organization. Crucially, compressive stimuli exhibit a strict dose-dependent regulatory effect on developmental checkpoints: 15% high-amplitude dynamic compression is essential for establishing and maintaining the early chondrogenic template [33], whereas reducing the strain to 10% acts as a temporal switch to drive the hypertrophic phase and subsequent osteogenic lineage commitment [34], [35].

As the organoid matures and models the vascular invasion phase of endochondral ossification, the mechanical niche must incorporate tensile and fluidic forces. Cyclic mechanical stretch guides mesenchymal fate decisions by activating the Smad signaling pathway to direct osteogenic lineage specification while restricting adipogenic trajectories [36], [37]. The sensitivity of these cells to such stretching cues is modulated by epigenetic regulators like Dnmt3b, which negatively regulates the Shh gene promoter [38], alongside specific miRNA expression profiles [39], [40]. Furthermore, the temporal pattern of these physical cues strictly dictates morphogenetic success. Intermittent stretch provides the necessary rhythmic cues to advance maturation, whereas continuous cyclic mechanical tension disrupts this progression by down-regulating RUNX2 expression and arresting the developmental process [41]. Ultimately, the introduction of interstitial fluid flow during the late stages of organoid development serves as the master mechanical switch for matrix mineralization. The sensing of fluid shear stress (FSS) is highly dependent on primary cilia and the mechanosensitive ion channels Piezo1 and TRPV4 [42], [43]. Flow-induced deflection of primary cilia opens Piezo1 channels, causing a rapid influx of extracellular Ca^2+^. Notably, the activation of Piezo1 extends beyond initiating intracellular calcium transients. It serves as a crucial driver for multicellular self-assembly. Recent evidence demonstrates that Piezo1 activation significantly upregulates the expression of key cellular adhesion molecules, specifically E-cadherin and N-cadherin, thereby accelerating the condensation of stem cells into tightly organized spheroids [44]. This mechanically-induced intercellular cohesion synergizes with the canonical Wnt signaling axis to rapidly augment the osteogenic capacity of the organoids. Subsequently, the intracellular calcium transient triggers the FAK-COX2 signal axis, inducing the secretion of prostaglandin E2 (PGE2) to finalize matrix mineralization [45], [46]. Beyond directly guiding osteogenesis, these dynamic physical forces—both compression and FSS—orchestrate the multicellular ecosystem by steering macrophage polarization from a pro-inflammatory M1 to an anti-inflammatory M2 phenotype [47], [48]. This mechanically-driven immunomodulation is vital for establishing a permissive osteoimmune niche, ensuring the synergistic self-organization and long-term functional maturation of the bone organoid.

In addition to direct mechanical loading, low-intensity pulsed ultrasound (LIPUS), as a kind of acoustic energy, has also attracted much attention in bone tissue engineering due to its non-invasive nature [49]. LIPUS usually operates at the frequency of 1-3 MHz and power of ∼30 mW/cm^2^, and acts on deep tissues through micro-vibration generated by acoustic radiation force [50]. Mechanistic studies have shown that LIPUS can effectively activate the Smad signaling pathway and up-regulate the expression of BMP-2 and its downstream transcription factor Osterix [50]. In addition, LIPUS can accelerate the process of bone development by activating SDF-1/CXCR4 signaling pathway [51] and Rho-COT/Tpl2-MEK-ERK signaling pathway [52] and simultaneously directing spatial migration and osteogenic lineage specification of MSCs. Han et al. [53] further showed that 20-min LIPUS can also upregulate IL-11 expression and activate the canonical Wnt/β-catenin pathway, forcefully steering BMSCs toward an osteogenic developmental trajectory while suppressing adipogenic drift. Interestingly, the signaling pathway activated by LIPUS showed cell type specificity: in BMSCs, it was mainly dependent on the JNK MAPK pathway, while in dental pulp stem cells (DPSCs), it was more dependent on the ERK1/2 pathway [54].

**Fig. 3:**
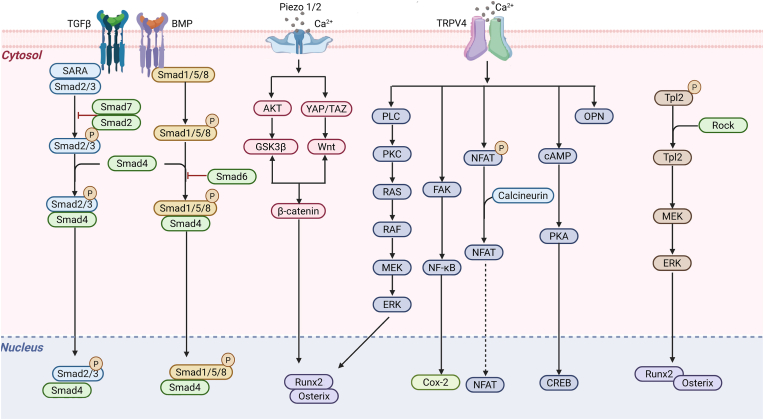
The regulatory mechanisms of various external active mechanical stimuli in bone remodeling. Superscript P: phosphorylation. Abbreviations: TGF-β: transforming growth factor-β; SARA: Smurf1 associated with RhoA; Rho: Ras homolog gene family; Smad: mothers against DPP homolog; BMP: bone morphogenic protein; AKT: serine/threonine kinase, protein kinase B; GSK3β: glycogen synthase kinase 3β; YAP/TAZ: Yes-associated protein/transcriptional coactivator with PDZ-binding motif; Wnt: wingless type; Runx2: runt-related transcription factor 2; TRPV4: transient receptor potential vanilloid type 4; PLC: phospholipase C-β; PKC: protein kinase C; RAS: rat sarcoma monomeric GTP-binding proteins; RAF: rat fibrosarcoma serin/threonine-protein kinase; MAPK: mitogen-activated protein kinase; MEK: MAPK kinase; ERK: extracellular signal-regulated kinase; FAK: focal adhesion kinase; NF-κB: Nuclear factor-κB; Cox-2: cyclooxygenase-2; NFAT: nuclear factor of activated T cells; cAMP: cyclic adenosine monophosphate; PKA: protein kinase A; CREB: cAMP response element-binding protein; OPN: osteopontin; Tpl2: tumor progression locus 2.

Therefore, when constructing bone organoids, static culture alone cannot activate these biochemical networks necessary for maintaining bone homeostasis, and the introduction of dynamic mechanical loading system is the key to achieve functional maturation of organoids. Fig. 3 and Table 1 provide a detailed summary of the signal transduction process of dynamic mechanical stimulation during bone development.

**Table 1:** The regulatory mechanisms of various external active mechanical stimuli in bone remodeling.

Stimulation Form	Signaling Pathway/Molecular Mechanism	Description	Ref.
Stretch	**Smad Signaling Pathway**	Promotes osteogenic lineage commitment and inhibits adipogenic differentiation.	[36], [37]
	**Shh Promoter/Dnmt3b**	DNA methyltransferase 3b (Dnmt3b) binds to the Shh promoter, negatively regulating sensitivity to stretch stimulation.	[38]
	**miRNA Regulation**	Downregulates miR-503-5p (promotes osteogenesis); miR-154-5p negatively regulates Wnt/PCP pathway.	[39], [40]
	**Wnt/PCP (Rhoa-Rock)**	Negatively regulated by miR-154-5p; inhibition suppresses osteogenesis in ADSCs.	[40]
	**ROS/AMPK-SIRT1**	Inhibits ROS (in young donors); activates AMPK-SIRT1 to lower intracellular ROS levels and promote osteogenesis.	[55], [56]
	**Runx2 Regulation**	Intermittent stretch promotes Runx2; Continuous cyclic mechanical Tension downregulates Runx2.	[41]
	*(General Mechanism)*	Proven effective in promoting osteogenic differentiation of MSCs from various sources.	[38], [57]
Compression	**BMP Upregulation**	Does not directly regulate Runx2 but induces osteogenesis via upregulation of BMP expression.	[32]
	**ERK1/2 Signaling Pathway**	Inhibition of ERK1/2 shifts cell fate from chondrogenesis to osteogenesis.	[33]
	**Immunomodulation/Macrophage Polarization**	Promotes macrophage polarization from M1 (pro-inflammatory) to M2 (anti-inflammatory); enhances anti-inflammatory properties.	[47], [58]
	*(Strain Magnitude Dependence)*	10% compression favors osteogenesis; 15% favors chondrogenesis; excessive compression weakens osteogenesis.	[34], [35]
	*(Autocrine Regulation)*	Context-dependent effects involving complex interactions between inflammatory cytokines and osteogenesis.	[48]
	*(General Mechanism)*	Can substitute for osteogenic induction medium to promote differentiation and mineralization.	[59]
Fluid Shear Stress	**Mechanosensitive Channels/Structures**	Initiates signal cascades via primary cilia, TRPV4, and Piezo1 ion channels.	[42], [43]
	**FAK-COX2 Signaling Pathway**	Triggers Ca^2+^ release, activating Akt, MAPK, and FAK. FAK is central, inducing COX2 and anti-inflammatory mediators.	[46]
	**Immunomodulation/Anti-inflammatory**	Wall Shear Stress (WSS) inhibits TNF-α synthesis and stimulates secretion of anti-inflammatory mediators (e.g., PGE2).	[45]
	*(General Mechanism)*	Important *in vivo* biophysical signal effective in promoting MSCs osteogenesis.	[60], [61]
LIPUS	**Smad Signaling Pathway**	Upregulates BMP-2 and downstream Osterix expression, promoting mineralization.	[50]
	**SDF-1/CXCR4**	Promotes osteogenic differentiation and cell migration.	[51]
	**Rho-Cot/Tpl2-MEK-ERK**	Promotes osteogenic differentiation and migration speed, accelerating bone repair.	[52]
	**Wnt/β-catenin (IL-11)**	Promotes IL-11 expression, activating Wnt/β-catenin pathway to promote osteogenesis and inhibit adipogenesis.	[53]
	**MAPK Family (ERK/JNK/p38)**	Cell-type dependent: DPSCs (ERK1/2); BMSCs (JNK).	[54]
	*(General Mechanism)*	Exogenous physical intervention showing significant effects in bone healing.	[49]

### 2.3 Spatial location-mediated cell interactions

Natural bone tissue is a highly ordered cellular society, and its homeostasis depends on the complex crosstalk among osteoblasts, osteocytes, osteoclasts and immune cells. This intercellular communication does not occur randomly, but depends on strict spatial isolation and signal gradients [62], [63]. As the commander of bone metabolism, osteocytes are deeply buried in the mineralized matrix and regulate cellular activities at the bone surface remotely by secreting signaling molecules. Under mechanical load, osteocytes reduce the secretion of sclerostin and relieve the inhibition of Wnt pathway to promote osteogenesis [64], [65]. However, in the state of mechanical unloading or injury, RANKL expression is up-regulated and RANKL/OPG ratio is changed to induce osteoclast precursor cell differentiation for bone resorption [30]. Such precise feedback regulation is extremely difficult to reproduce in cell masses in pure mixed cultures because the necessary spatial structure to establish a stable paracrine gradient is lacking [66]. In addition, the microenvironment of bone organoids is deeply regulated by the immune-bone axis. The polarization of immune cells, especially macrophages determine bone growth and development. Studies have shown that moderate mechanical stimulation can guide the polarization of macrophages to the M2 type conducive to osteogenesis by regulating the secretion of cytokines, and synergetically promote bone regeneration by secreting factors such as PGE2 [47], [58]. However, in the co-culture model without engineering guidance, different types of cells often grow disorderly and fail to establish an ordered structure like osteon, resulting in disordered signaling pathways. Therefore, the construction of bone organoids must rely on engineering methods to provide a spatial template to restrict self-assembly to a specific structural framework, so as to guide the chaotic cell population into a bionic tissue with ordered structure and complex communication functions.

### 2.4 Future perspectives on integrating engineering strategies into 3D bone organoid

In the specific context of engineered bone organoids, which fundamentally rely on macroscopic 3D porous templates rather than simple scaffold-free spheroids, integrating these responsive materials holds the profound potential to actively upgrade the entire biofabrication pipeline. During the initial seeding and colonization phase, thermo- or photo-responsive hydrogels coating the intricate 3D pore struts could undergo targeted volumetric contraction upon stimulation. This active topological morphing might physically trap and compact the infiltrated stem cells within the local meso-pores, effectively driving mesenchymal condensation directly within the spatial confines of the geometric framework.

As the construct advances into the early lineage commitment phase, electro-responsive 3D templates (e.g., piezoelectric ceramics or polymers) could harness endogenous cellular mechanobiology. As cells spread and generate traction forces across the porous struts, the template would spontaneously polarize to generate highly localized micro-currents, potentially driving osteogenic and osteoimmune differentiation across the expansive internal surface area without the need for external wiring. When scaling up the volumetric construct to clinically relevant macroscopic dimensions, magneto-responsive elements embedded within the 3D template network might become vital to overcome the diffusion limit. Actuation via external magnetic coils could induce periodic micro-deformations throughout the structural matrix, effectively functioning as an intrinsic “mechanical pump”. This internal actuation is anticipated to drive convective fluid flow through the complex multi-porous network, mitigating core necrosis while spatially guiding the deep invasion of co-cultured endothelial networks. Finally, to expedite the critical global biomineralization phase, acousto-responsive 3D templates could be stimulated with LIPUS. The acoustic waves would seamlessly penetrate the macroscopic porous architecture, interacting with the template's stiffening matrix to trigger widespread, synchronized intracellular calcium influx. This synergistic approach is expected to drastically accelerate matrix mineralization across the entire engineered organoid, ultimately culminating in a mature, volumetrically stable macroscopic bone organoid.

Looking beyond the active physical actuation of these templates, the ultimate functional maturation of bone organoids would necessitate the integration of smart, stimuli-responsive degradable materials. To achieve true organ-level autonomy, these engineered architectures must function as transient cues rather than permanent structural fixtures. Prospectively, programming the degradation kinetics to perfectly synchronize with endogenous ECM deposition could prevent the residual rigid template from shielding necessary mechanotransduction. Furthermore, the degradation process itself might be engineered as an active biological regulator; for instance, the controlled release of bioactive ions during template erosion could synergize with external physical fields to continuously enhance downstream signaling. Ultimately, transitioning towards fully degradable responsive systems is expected to eliminate the interference of residual inorganic nanoparticles, thereby ensuring the high biophysical fidelity of these organoids when deployed as *in vitro* disease models for pharmacological screening.

## 3 Morphogenetic templates design for engineered bone organoids

In the construction of bone organoids, three-dimensional scaffolds have evolved beyond being mere passive carriers for cell adhesion, instead, they serve as physical templates for morphogenesis. Drawing upon the design insights from conventional bone tissue engineering scaffolds, this chapter integrates the unique characteristics and developmental processes of bone organoids to systematically elaborate on the design criteria for engineered bone organoid templates, focusing on three key dimensions: mechanical biomimicry, mass transfer optimization, and topological guidance.

### 3.1 Porous structure

Unlike soft tissue organoids, the construction of bone organoids faces a more severe challenge of material transport, which is not only attributed to the increase in tissue size, but also the unique mineralized matrix of bone tissue. Classical theory states that when the diameter of avascular cell mass exceeds 200-400 μm, its core region will be significantly deficient in oxygen and nutrients, leading to the phenomenon of “core necrosis” [67], [68]. This biophysical process can be precisely described by Fick's first law of diffusion:

(2) $$J=-D_{eff}\frac{dc}{dx}$$

Where *J* is the diffusion flux, *dc/dx* is the concentration gradient, *D*_*eff*_ is the effective diffusion coefficient of the fluid, and *ε* is the porosity of the porous structure. *D*_*eff*_ is the key variable that determines the transmission efficiency. However, as bone organoids mature and become mineralized, hydroxyapatite (HA) crystals are tightly packed in the matrix, resulting in a sharp decrease in *ε* and a consequent decrease in *D*_*eff*_. At the same time, the high metabolic activity of osteoblasts [69], [70] further aggravates oxygen consumption and accelerates oxygen deprivation in the central region of the organoid. Natural bone solves the physical contradiction through the Haversian system, an elaborate microtubule network. However, cells cannot spontaneously assemble such long-range and penetrating channels *in vitro*. Therefore, the introduction of engineered morphogenetic templates became necessary to overcome the physical limitations. By designing porous scaffolds (pore size >100 μm) [71] with high connectivity (porosity 50%-80%) [72], artificial mass transfer channels can be established at the microscopic scale to overcome the failure of Fick's law in dense mineralized tissues, so as to maintain the survival and function of large-scale bone organoids. Table 2 summarizes the effects of pore characteristics on bone development outcomes (see Table 3).

**Table 2:** Effects of pore size and porosity on the function of traditional bone scaffolds.

Pore Size	Porosity	Effects	Cell type	Reference
<10 μm	-	Restricts migration but exponentially increases specific surface area, accelerating protein adsorption and >1.5-fold increase in early cell viability.	hMSCs	[73]
≤100 μm	-	Prone to fibrous tissue encapsulation, hindering vascular network assembly and cell-matrix crosstalk.	-	[71]
200–300 μm	-	Restricted spatial confinement induces MSCs toward chondrogenic lineages, establishing a cartilage template rather than direct ossification.	BMSCs	[74]
>300 μm	-	Conducive to robust vascularization and direct intramembranous osteogenesis.	-	[75]
325 μm		Exhibited the highest cell attachment and a significantly higher proliferation rate at 7 days.	MC3T3-E1 (Murine preosteoblasts)	[76]
500 μm	-	Provided mechanical parameters highly compatible with natural bone.	MG-63 (Human osteosarcoma cell line)	[77]
600 μm	∼70%	Exhibited mechanical properties closest to natural bone.	-	[78]
600 μm	-	Demonstrated the highest mechanical integration strength of the newly formed bone network.	*In vivo* only	[79]
650 μm	-	Most conducive to bone ingrowth.		[80]
1000 μm	77%	More conducive to initial cell adhesion and spreading.	MG-63	[77]
	Summarization			
100–900 μm	50%–80%	Commonly used range.	-	[72], [81]
500–700 μm	60%–70%	Exhibited the best mineralized tissue infiltration performance, and achieved the best structural integration between the template and the newly formed bone matrix.	-	[82], [83]
-	>65%	Facilitates deep ECM permeability and vascular network ingrowth, providing expansive interfaces for cell-ECM dynamic reciprocity.	MC3T3-E1	[76]
-	<65%	Provides rigid mechanical boundary conditions but restricts deep cell migration, often stalling the organoid maturation process.	MC3T3-E1	[76]

**Table 3:** Common TPMS constructor functions.

Type	Function
Primitive	cosx+cosy+cosz=C
Diamond	cosxcosycosz−sinxsinysinz=C
Gyroid	sinxcosy+sinycosz+sinzcosx=C
I-WP	2(cosxcosy+cosycosz+coszcosx)−(cos2x+cos2y+cos2z)=C
F-RD	cos2xsinycosz+cosxcos2ysinz+sinxcosycos2z=C
Neovius	3(cosx+cosy+cosz)+4cosxcosycosz=C

### 3.2 Bionic hierarchical structure

Natural bone is a composite material with a multi-level layered structure. From nanoscale mineral deposits to micrometer-scale bone tubular networks, and then to macroscopic cortical and cancellous bone divisions, this cross-scale orderliness jointly endows bone with excellent mechanical properties and biological functions. Therefore, the introduction of hierarchical gradient design in the construction of bone organoids, through the carefully designed combination of multi-level pore size, porosity and pore geometry, can not only synergistically optimize the mechanical properties, permeability and biological activity of the morphogenetic templates, but also accurately simulate the structural heterogeneity of the *in vivo* environment [84]. This bionic design can directly influence cell adhesion, migration, proliferation and osteogenic differentiation behaviors in *vitro* models by regulating the interaction between cells and ECM and activating different intracellular signaling pathways, thereby acting as a geometric orchestrator for multicellular self-organization and establishing a highly realistic microenvironment for bone organoids. The Gibson-Ashby model provides a theoretical basis for the mechanical design of porous scaffolds. For open-cell foam structures, the relative elastic modulus and relative density follow a power-law relationship:

(3) $$\frac{E}{E_{s}}=C{\left(\frac{\rho }{\rho_{s}}\right)}^{n}=C\cdot {\left(1-\varepsilon \right)}^{n}$$

**Fig. 4:**
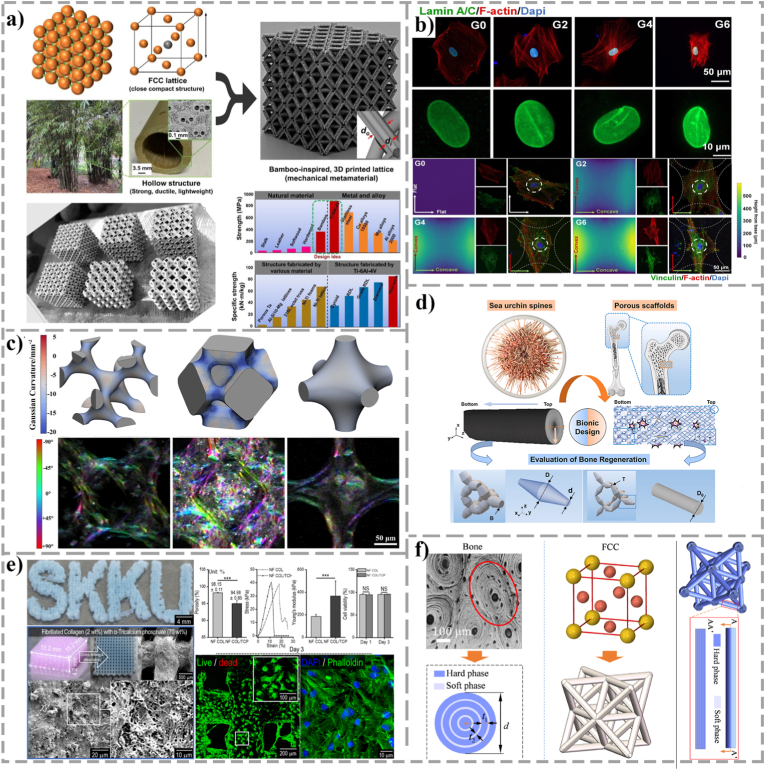
Application of mechanical metamaterials and layered gradient structures in bone tissue engineering scaffolds. (a) Face-Centered Cubic (FCC) lattice inspired by the high-strength and high-toughness hollow bamboo structure. A series of hollow isotropic mechanical metamaterials have been built using high-fidelity SLM technology, successfully achieving the unification of lightweight and high strength (Adapted with permission from Ref. [87]. Copyright 2022, Elsevier.) (b) The triply periodic minimal surface (TPMS) scaffold successfully guided cells towards osteogenesis through directional bending at the cell level and showed huge but quantifiable improvements in bone regeneration (Adapted from Ref. [91] under a CC BY 4.0 license.) (c) Surface curvature can be used as a morphological clue to affect cell proliferation, growth and osteogenic differentiation (Adapted with permission from Ref. [92]. Copyright 2025, IOP Publishing.) (d) A five-mode pentamode metamaterials biomimetic scaffold based on the microstructure of sea urchin vertebrae (Adapted from Ref. [88] under a CC BY 4.0 license.) (e) Layered nanofiber collagen scaffolds with different pillar thicknesses, the structure containing both macropores, micropores and nanofiber pillars. The nanofiber structure provides a huge specific surface area, significantly enhances the adsorption of multiple key ECM proteins, and supports the unipolar extension of the cytoskeleton, thereby facilitating the extension and contraction of the cellular structure (Adapted with permission from Ref. [89]. Copyright 2018, American Chemical Society.) (f) The biomimetic design strategy of combining a light weight and high specific strength metal FCC lattice structure with a bone-like concentric structure with high toughness has greatly improved the toughness of the structure (Adapted with permission from Ref. [90]. Copyright 2022, Elsevier.)

Here *E* and *ρ* are the modulus and density of the porous scaffold, *E*_*s*_ and *ρ*_*s*_ are the modulus and density of the matrix material, and *ε* is the porosity, respectively. Based on this model, templates with gradients of modulus spanning several orders of magnitude can be fabricated by spatially continuously adjusting the porosity or cellular structure of the templates. By integrating macroporous (>100 μm) and mesoporous (50-100 μm) structures into the scaffold, Xu et al. [85] significantly enhanced the recruitment ability of BMSCs and up-regulated the expression of osteogenic markers (OPN, BMP-2, and RUNX2). The importance of topological gradients was further validated by Lei et al. [86], who engineered hydroxyapatite templates mirroring the hierarchical pore characteristics of native bone. Rather than merely serving as structural fillers, these biomimetic geometric cues rapidly orchestrated cellular self-organization and accelerated the deposition of a mineralized matrix, mimicking the intrinsic developmental trajectory of native bone. In addition, Zhang et al. [87], inspired by the hollow bamboo structure with high strength and toughness, used high-fidelity SLM (Selective laser melting) technology to construct a series of hollow isotropic mechanical metamaterials based on face-centered cubic (FCC) lattice, and successfully achieved the unified high strength and high material transfer efficiency (Fig. 4(a)). Zhang et al. [88] developed the pentamode metamaterials biomimetic structure (Fig. 4 (d)) by SLM technology based on the microstructure of sea urchins vertebrae. Featuring a gradient porosity and a tapered topology, this structure actively resolves the core nutrient deprivation typically plaguing conventional static cultures. Its hierarchical pore distribution replicates the native skeletal niche, providing equivalent mechanical boundary conditions while significantly facilitating deep cellular infiltration and interconnected network formation. Consequently, it effectively drives robust morphogenetic progression and autonomous biomineralization throughout the entire engineered construct. Resolving the inherent biophysical antagonism between mechanical stiffness and fluid permeability via such hierarchical gradients is a prerequisite for scaling up macroscopic bone organoids without succumbing to core necrosis. From the perspective of fiber topology, Lee and Kim [89] prepared a layered nanofibrous collagen scaffold with different pillar thicknesses, which simultaneously contained macropores, micropores and nanofiber pillars (Fig. 4 (e)). *In vitro* experiments showed that the nanofiber structure provided a large specific surface area, significantly enhanced the adsorption of several key ECM proteins such as fibronectin and vitronectin. Geometrically, it directed the monopolar extension of the cytoskeleton, translating physical contact guidance into dynamic cytoskeletal contractility and mechanotransduction. Finally, Wei et al. [90] proposed a multi-biomimetic strategy, ingeniously combining the lightweight and high-specific strength metallic FCC lattice structure with the bone-like concentric structure with high toughness (Fig. 4(f)). By adjusting the ratio of soft and hard phases in the structure, the toughness of the scaffold can be improved while providing suitable substrate stiffness for different cell types, so that the ordered self-assembly of different cell types within a specific structural framework can be induced.

### 3.3 Optimization design of material transfer performance

Mass transport efficiency dictates whether bone organoids can surpass size limitations and avoid core necrosis. Crucially, optimizing mass transport necessitates a multi-scale approach, encompassing both the microscopic diffusion within the cellular spheroids and the macroscopic perfusion through the template. At the microscopic scale, nutrient diffusion inside the dense cell mass is strictly limited by Fick's law, making it exceptionally difficult to maintain the survival of avascular tissues with a diameter exceeding 400 μm [67], [68]. According to Yuan et al. [93], intra-spheroid oxygen gradients are highly size-dependent. While excessive aggregation sizes inevitably lead to severe hypoxia and pathological core necrosis, precisely controlled spatial confinement can induce moderate physiological hypoxia within the spheroid core. This localized, controlled hypoxic niche acts as a potent trigger, activating HIF-1α pathways and robust VEGF secretion to orchestrate the intrinsic angiogenic potential of the organoid. Therefore, the morphogenetic template must first impose stringent geometric boundaries to restrict spheroid dimensions within an optimal bioengineered window, striking a delicate balance between deep cellular viability and pro-angiogenic functionality.

Beyond imposing micro-scale spatial boundaries, the engineered template must simultaneously optimize macroscopic transport to sustain these self-assembled spheroids. It transforms pure passive diffusion into “diffusion-convection” coupled transmission by introducing a highly interconnected porous network. To quantify this macroscopic material transport efficiency of the template, the concept of permeability from fluid mechanics is introduced. Permeability is an intrinsic physical property that measures the ability of a porous medium to allow fluid to pass through its interconnected pores, which can be mathematically calculated using the Kozeny-Carman equation:

(4) $$k=\frac{\varepsilon^{3}}{K_{c}{\left(1-\varepsilon \right)}^{2}{S_{v}}^{2}}$$

Where *k* is the permeability, *ε* is the porosity, *S*_*V*_ is the specific surface area, and *K*_*c*_ is the empirical constant related to the topology of the pore. According to the K-C equation, the permeability *k* is proportional to the porosity *ε*. Studies have shown that in order to obtain a permeability similar to that of natural cancellous bone [94] (2.8 × 10^−10^ m^2^ to 1.3 × 10^−8^ m^2^), the porosity of the template usually needs to be maintained between 60% and 90%. However, too high porosity can significantly reduce the mechanical strength of the template (Equation (3)). By designing the topological morphology of porous templates, the permeability can be improved by reducing *K*_*c*_ without compromising the mechanical strength. For example, Foroughi et al. [95] optimized the porosity of a FCC scaffold by integrating mathematical representations, finite element analysis, and computational fluid dynamics (CFD) simulations, thus finding the equilibrium point between the Young's modulus and permeability of the scaffold. Wang et al. [96] reduced the stress concentration of the diamond structure and improved the permeability by adjusting different microscopic parameters of the scaffold. Li et al. [97] adopted the method of multi-objective optimization to optimize the structure of TPMS, so that the permeability of the structure could be changed from 0.12 × 10^−8^ m^2^ to 0.83 × 10^−8^ m^2^ under the condition of the same Young's modulus. Although high permeability is beneficial for macroscopic transport of nutrients, the hydrodynamic environment inside the scaffold, especially WSS, has a decisive influence on cell fate in dynamic culture environments. In a parallel-plate flow chamber or microfluidic chip, the laminar shear force *τ* is proportional to the volume flow rate *Q*, following the formula

(1) $$\tau =\frac{6\mu Q}{\omega h^{2}}$$

Here, *μ* is the hydrodynamic viscosity, *ω* is the width of the flow chamber, and *h* is the height of the flow chamber. Kim et al. [98], using a bone organ-on-a-chip system integrated with TPMS scaffold, found that although the high-porosity TPMS scaffold had extremely high permeability, its internal flow rate was too fast under dynamic perfusion, resulting in WSS beyond the physiological window suitable for osteogenesis, thus inhibiting late mineralization deposition. On the contrary, the template with suitable porosity produced a uniform and moderate shear stress distribution on the template surface through optimized curvature and moderate flow resistance, which significantly promoted collagen generation and matrix mineralization. This finding suggests that when designing the mass transfer performance of scaffolds, we should not simply pursue the maximum permeability, but must combine CFD simulation to find the best topological balance between efficient material transfer and appropriate mechanical stimulation.

### 3.4 Triply periodic minimal surface (TPMS) structure

The microscopic topology of scaffolds is an important physical clue to regulate cell morphology, arrangement, and differentiation fate. Among the many topological configurations, the TPMS structure has attracted wide attention due to its high similarity with the topological morphology of natural trabecular bone. Since the TPMS structure is composed of smooth and continuous curved surfaces, it is significantly superior to the traditional solid porous structure in terms of stress transmission, which can achieve effective structural lightweight and mechanical behavior matching with natural bone, and promote the osteogenic differentiation of stem cells [99]. Besides, the TPMS structure has unique surface curvature characteristics, which can effectively affect the osteogenic behavior of cells. Suppose there is a smooth surface S defined on a planar region Ω that can be represented by the function *z = f* (*x,y*), then a surface S satisfying the following partial differential formula is called TPMS:

(5) $$\left(1+f_{y}^{2}\right)f_{xx}-2f_{x}f_{y}f_{xy}+\left(1+f_{x}^{2}\right)f_{yy}=0$$

Where *f*_*x*_ is the partial of function *f* to the parameter *x*, *f*_*y*_ is the partial of function *f* to the parameter *y*, *f*_*xx*_ is the partial of *f*_*x*_ to the parameter *x*, *f*_*xy*_ is the partial of *f*_*x*_ to the parameter *y*, *f*_*yy*_ is the partial of *f*_*y*_ to the parameter *y*. Among them, the commonly used TPMS equations are shown in the following table.

Here, *C* is the threshold constant for controlling porosity. This mathematical continuity endows the scaffold with extremely smooth surface features, eliminating the stress concentration points in traditional truss structures and facilitating cell adhesion and spreading. Moreover, this continuity and smoothness enable TPMS structures to have a significantly lower *K*_*c*_ than traditional truss structures under the same porosity conditions [100], thereby effectively enhancing permeability without compromising the mechanical properties of the scaffold [101]. This characteristic not only facilitates the rapid transport of nutrients but also more effectively transmits fluid shear stress to the cell surfaces deep within the scaffold, activating mechanical transduction signals [102].

Consequently, TPMS structures have emerged as highly potent morphogenetic templates. Yang et al. [91] fabricated β-tricalcium phosphate TPMS structures with different Gaussian curvatures through SLA printing and high-temperature sintering. Compared with traditional structures, TPMS structures induce the directional bending of cells through surface curvature, promoting the expression of Lamin A/C. This mechanical confinement activates the intracellular FAK/MAPK pathway, actively driving the osteogenic developmental trajectory rather than merely supporting cell survival (Fig. 4(b)). Exploring the micro-scale, Han et al. [92] used two-photon 3D printing to fabricate TPMS structures with different curvature distributions. Their findings delineated a dual-path morphogenetic mechanism where surface curvature dictates organoid fate. On one hand, the zero mean curvature inherent to TPMS significantly enhances internal fluid permeability and metabolic exchange, mitigating the diffusion limitations within dense organoids. On the other hand, the widely distributed negative Gaussian curvature on the TPMS acts as a physical contact guidance cue, forcing the inoculated cells to align and extend along the main direction of the surface. This reorganization of the cytoskeletal tension induced by the microscopic geometric morphology can directly translate into biochemical signals, significantly upregulating the expression of key osteogenic genes such as RUNX2 (Fig. 4 (c)). In addition to its excellent mass transfer performance, another core advantage of the TPMS structure lies in its ability to precisely replicate the complex mechanical anisotropy of natural bone. Traditional trusses or lattice supports often exhibit uniform tensile and compressive properties, which are fundamentally different from the nonlinear responses of natural bone under different loading conditions. The latest research by Karali et al. [103] indicates that the 3D-printed titanium alloy scaffold based on the Diamond topology design can reproduce the unique tensile-compressive asymmetry of cortical bone, with its tensile modulus (∼38-55 GPa) significantly higher than the compressive modulus (∼14-20 GPa). This inherent mechanical asymmetry, derived strictly from the TPMS pore topology, functions directly as a physical induction template, providing a highly biomimetic mechanical microenvironment that guides pre-osteoblast self-organization.

Therefore, the TPMS structure is not only a physical carrier for cells, but also an intelligent microenvironment integrating efficient mass transfer and active induction functions, and is an ideal topological choice for constructing functionalized bone organoids.

The advanced topological structure precisely designs the spatial layout, creating a highly biomimetic static physical microenvironment for bone-like organs. However, bone operates in a dynamic mechanical environment, and its development, homeostasis, and repair are precisely regulated by continuous changing biomechanical signals. The current scaffold paradigm based on static structures faces a fundamental challenge: the mechanical properties of these scaffolds are fixed after implantation, unable to simulate the dynamic evolution of *in vivo* biomechanical signals, and difficult to provide differentiated mechanical support at different stages of tissue regeneration. This leads to a static trade-off between mechanical properties and biological functions that is difficult to overcome: an optimized structure designed for initial cell adhesion and vascularization may not be suitable for the mechanical environment required for bone matrix maturation and mineralization in the later stages. To break through this limitation, future research directions are shifting from constructing static physical supports to developing intelligent scaffolds that can actively simulate the dynamic biomechanical environment *in vivo*. Mechanical response materials can be the core for achieving this leap. These materials (such as piezoelectric, magnetostrictive, and acoustic response materials) can convert external energy into changes in the mechanical signals within the scaffold, thereby enabling in situ, dynamic, and controllable mechanical stimulation of cells. This article will detail the characteristics, response mechanisms, and application prospects of these mechanical response materials in the construction of bone-like organs.

## 4 Construction strategy and biological effects of dynamic microenvironment

Bone is not only a physical support structure, but also a physiological system in a highly dynamic mechanical environment. Its development, homeostasis maintenance, and remodeling processes are always precisely regulated by fluid shear, mechanical load, and bioelectrical signals. Although traditional static 3D culture provides a basic spatial boundary, it is difficult to reproduce this complex dynamic physiological microenvironment. As a result, *in vitro* cultured bone organoids are often difficult to form mature mineralized structures and functions due to the lack of necessary physical instructions. Therefore, the key to constructing functional bone organoids is to introduce a dynamic stimulation system that can simulate the mechanical environment *in vivo*.

### 4.1 Intelligent response to stimuli mediated by materials

Despite the effectiveness of external physical field stimulation, complex 3D organoid cultures often face problems of deep signal attenuation or cumbersome device connectivity. To solve this limitation, intelligent responsive materials have been introduced into the next generation of bone organoid templates. Transitioning from passive structural fillers to active transducers, these materials sense external wireless fields (magnetic, acoustic, or light) and locally convert them into *in-situ* electrical, mechanical, or thermal instructions, thereby orchestrating multicellular self-organization from the inside out.

#### 4.1.1 Electric responsive material

The core of electrically responsive materials lies in their piezoelectric effect or conductivity. The piezoelectric properties of bone enable it to convert mechanical stress into endogenous electrical signals, which drive embryonic skeletal development and lifelong remodeling [124], [125]. Piezoelectric materials will produce electric polarization when subjected to mechanical strain, forming an electric potential on the surface of the material. Conductive materials, such as polypyrrole, can provide a channel for the cell to transfer electrons or undergo physical deformation under an applied electric field. Crucially for bone organoids, these localized electrical cues directly activate voltage-gated calcium channels on the cell membrane, promoting early-stage mesenchymal condensation and directing the developmental trajectory without the need for bulky external electrodes.

**Fig. 5:**
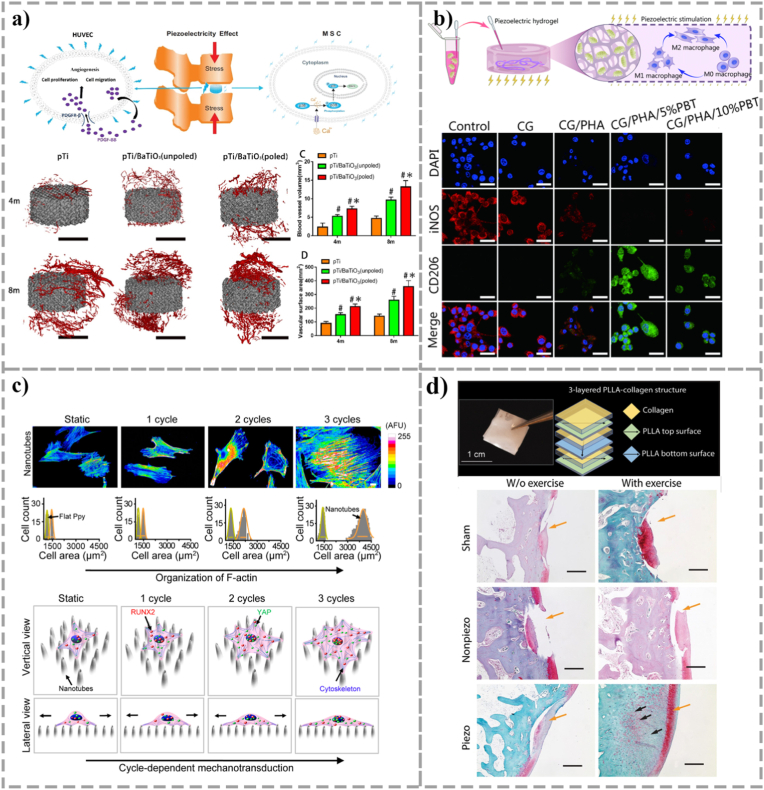
Application of electrically responsive biomaterials in bone tissue engineering. (a) The BTO-coated scaffold prepared on a porous titanium alloy (Ti6Al4V) substrate simultaneously promotes the osteogenic differentiation of MSCs and the vascularization process of human umbilical vein endothelial cells through piezoelectric effect, and its effects on enhancing osteogenesis and angiogenesis were verified in a sheep spinal fusion model. Scale bar: 6 mm. (Adapted with permission from Ref. [105]. Copyright 2020, American Chemical Society.) (b) The polydopamine-modified HAP/BTO porous hydrogel scaffold not only creates a piezoelectric microenvironment, but also plays an immunoregulatory role by regulating the M2 polarization of macrophages, synergistically promoting bone regeneration and angiogenesis. Scale bar: 25 μm. (Adapted from Ref. [106] under a CC BY 4.0 license.) (c) Polypyrrole (PPy) nanotubes can achieve reversible morphological transformation through electrical stimulation, triggering mechanical transduction gene expression and osteogenic biomarker production in only 3-5 driving cycles (40 min per cycle). (Adapted with permission from Ref. [111]. Copyright 2017, American Chemical Society.) (d) PLLA and collagen composite piezoelectric structure successfully promoted osteochondral regeneration of rabbit knee joints through mechanical activation, and solve the non-degradable problem. Scale bars: 500 μm. (Adapted with permission from Ref. [110]. Copyright 2022, The American Association for the Advancement of Science.)

Based on this principle, researchers have developed a variety of piezoelectric and conductive biomaterials that mimic the electrical properties of natural bone tissue. In the field of piezoelectric ceramics, lead-free materials such as barium titanate (BTO) and zinc oxide (ZnO) have received much attention due to their good biocompatibility [126], [127]. Injectable gelatin methacryloyl (GelMA)/BTO hydrogel developed by Roldan's group [104] has been shown to provide micro-electrical cues to enhance the osteogenic commitment of BMSCs. The BTO-coated scaffold prepared by Liu et al. [105] on a porous titanium alloy (Ti6Al4V) substrate simultaneously promoted the osteogenic differentiation and angiogenesis process of MSCs through the piezoelectric effect (Fig. 5 (a)). Wu et al. [106] used polydopamine modified HAP/BTO porous hydrogel scaffolds, which can not only create a piezoelectric microenvironment, but also play an immunomodulatory role by regulating the M2 polarization of macrophages, which is essential for establishing a permissive microenvironment for organoid maturation (Fig. 5 (b)). In the realm of piezoelectric polymers, PVDF and its copolymer PVDF-TrFE offer essential flexibility [128]. Composite templates, such as PVDF/TiO_2_ [107] and PVDF-TrFE/BNNTs [108], have been shown to accelerate osteoblast maturation and ECM deposition. Liu et al. [110] made them into piezoelectric nanofiber scaffolds, which successfully provided sustained mechanobiological activation for osteochondral interface formation (Fig. 5 (d)). In addition, Wei et al. [111] found that conductive polymers like polypyrrole (PPy) can undergo reversible morphological transformations under electrical fields, translating transient electro-mechanical cues into sustained osteogenic signaling (Fig. 5 (c)).

The significant advantage of electro-responsive materials is that their signals are highly consistent with the natural physiological activities of bone tissue, which can directly interfere with the electrophysiological process of cells. Moreover, piezoelectric materials can realize the self-driven conversion of mechanical energy to electric energy without external continuous energy supply. However, the development of this field still faces serious challenges. At present, most high-performance piezoelectric ceramics (such as BTO) and polymers (such as PVDF) are difficult to biodegrade in the physiological environment, and the biosafety risks caused by their long-term retention cannot be ignored. At the same time, whether the long-term stability of the piezoelectric effect of materials can be sustained in the complex biological environment *in vivo*, and how to achieve uniform and precise regulation of electrical signal parameters within 3D bone organoids are still problems that must be solved.

#### 4.1.2 Magnetic responsive material

Magnetically responsive biomaterials are usually composed of magnetic nanoparticles (MNPs) embedded in biocompatible matrices. Their unique deep-tissue penetration offer a profound advantage for bone organoid biofabrication [129], [130]. Unlike fluid shear stress in macro-bioreactors, which often diminishes at the organoid core, external magnetic fields can uniformly penetrate dense mineralized matrices to deliver wireless mechanotransduction.

**Fig. 6:**
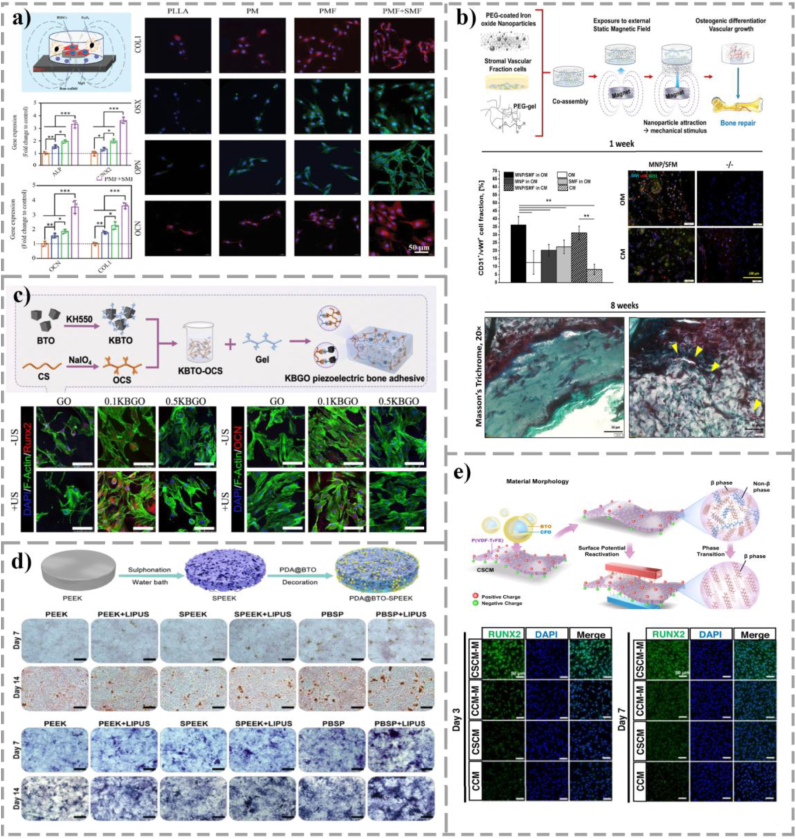
Application of magnetic and ultrasonically responsive biomaterials in bone tissue engineering. (a) Magnesium-loaded magnetic bone scaffold. Under the action of SMF, the scaffold can accelerate the uptake of Mg^2+^ in the surrounding microenvironment. This enhanced Mg^2+^ influx leads to improved cellular activity and increased expression of osteogenesis-related genes. (Adapted with permission from Ref. [113]. Copyright 2023, John Wiley and Sons.) (b) PEG/MNP hydrogel system, which uses gradient SMF to drive MNPs to make them controllable within the hydrogel. Cells inside the magnetically driven hydrogel show increased metabolic activity, promoting osteogenesis and angiogenesis of the cells. Scale bars: white = 50 μm, black = 50 μm. (Adapted with permission from Ref. [118]. Copyright 2019, Elsevier.) (c) An injectable ultrasound-responsive nanocomposite hydrogel that is shape-compliant, highly bone-viscous. This ultrasound-responsive nanocomposite hydrogel enhances osteogenic differentiation of BMSCs by increasing Ca^2+^ inflow and upregulating PI3K/AKT and MEK/ERK signaling pathways. Scale bar: 100 μm. (Adapted from Ref. [122] under a CC BY 4.0 license.) (d) PEEK (PBSP), mediated by polydopamine (PDA), exhibits a piezoelectric effect under low-intensity pulsed ultrasound (LIPUS), promotes osteogenesis by modulating Piezo1, induced Ca^2+^ influx and the Akt/GSK3β/β-catenin pathway. Scale bar: 200 μm. (Adapted from Ref. [123] under a CC BY 4.0 license.) (e) CoFe2O4@BTO/PVDF-TrFE core-shell structural membrane. This charged membrane covers the bone defect, retaining space for healing and stimulating osteogenesis and blood vessel formation. Scale bar: 50 μm. (Adapted from Ref. [120] under a CC BY 4.0 license.)

Of all types of excitation magnetic fields, static magnetic field (SMF) has been the most extensively studied. Under SMF, MNPs can generate magnetic torque, exert mechanical stimulation on the cell membrane or magnetic receptors in cells, therefore affect ion channels [112], [113]. For example, Yan et al. [113] incorporated MNPs into PLLA/MgO composites accelerates Mg^2+^ uptake under an 80 mT SMF, which promotes intracellular mineralization cascades [114] and enhances overall organoid viability (Fig. 6 (a)). At the nanoscale, MNPs within PLGA matrices [117] induce physical membrane deformations, directly upregulating the critical mechanosensitive gene Piezo1. Furthermore, gradient SMFs can be utilized to spatially pattern MNPs within PEG hydrogels developed by Filippi et al. [118], guiding the spatiotemporally organized co-culture of endothelial and osteogenic lineages, thereby resolving the architectural complexity of vascularized bone organoids (Fig. 6 (b)). Alternatively, under an alternating magnetic field (AMF), magneto-controlled templates undergo periodic physical movements, generating internal shear stress and compressive strains that physically massage the seeded cell spheroids [119], [131]. When combined with piezoelectric materials, MNPs can respond to the applied magnetic field and undergo deformation, thereby initiating the mechanical transduction process within the cells [132]. This deformation can further activate the intracellular signaling pathways in bone development. Liu et al. [120] prepared a CoFe2O4@BTO/PVDF-TrFE core-shell structure membrane, which ingeniously coupled magnetic and piezoelectric effects. These membranes convert remote AMF signals into internal electrical stimuli, driving robust bone organoid maturation even in inhibitory inflammatory niches (Fig. 6 (e)). Electromagnetic field (EMF) therapy, due to its non-invasive nature and safety, has increasingly become an important treatment method in the field of orthopedics. Its effectiveness in promoting the osteogenic differentiation of MSCs has been fully verified [133], [134]. Aldebs et al. [135] designed a synergistic system combining pulsed EMF with superparamagnetic iron oxide nanoparticles (SPIONs), effectively triggers early osteogenic lineage commitment in human adipose-derived stem cells (hASCs) into the osteoblast lineage. Under the action of SMF, SPIONs endow poly(lactic-co-glycolic acid) (PLGA) microspheres with special magnetism, promoting the differentiation of BMSCs and upregulating the expression of ALP, COL1, OPN, and OCN *in vivo* [136].

The core appeal of magnetic response technology is its excellent tissue penetration capability, allowing non-invasive remote precise modulation of deep tissues with high spatial and temporal resolution. However, this prospect also comes with clear constraints. The long-term retention, potential biodegradability, immunogenicity and toxicity of magnetic nanoparticles are the main obstacles to their clinical transformation. In addition, it is technically difficult to ensure uniform distribution of magnetic field gradients and resulting mechanical stimuli in complex 3D organoids, and there are still challenges in miniaturization and popularization of high-magnetic field devices suitable for long-term and safe clinical applications.

#### 4.1.3 Ultrasonic responsive material

Ultrasound, especially LIPUS, is an effective mechanical stimulation in itself. When combined with the material, the response mechanism is mainly reflected in two aspects: first, the mechanical vibration and acoustic cavitation effects of the ultrasonic wave act directly on the cells. The second is to activate piezoelectric materials to generate electrical signals to achieve acoustically electric conversion. This synergistic effect can jointly regulate intracellular Ca^2+^ influx and key signaling pathways such as PI3K/AKT and MEK/ERK [122], [123].

Ultrasonic stimulation is often combined with piezoelectric materials to promote bone remodeling in practical applications. Tandon et al. [121] demonstrated that integrating LIPUS with PVDF templates exponentially amplified alkaline phosphatase (ALP) activity ∼ 3-times compared to static cultures. Advancing this concept, Zhou et al. [122] developed a shape-adaptive, highly bone-cohesive, injectable ultrasound-responsive nanocomposite hydrogel through the dynamic covalent cross-linking of piezoelectric nanoparticles and a biopolymer hydrogel network. This ultrasound-responsive nanocomposite hydrogel enhanced osteogenic differentiation of BMSCs by increasing calcium influx and upregulating PI3K/AKT and MEK/ERK signaling pathways (Fig. 6 (c)). Li et al. [123] exploited the piezoelectric effect of BTO exhibited under LIPUS to develop an ultrasonic responsive PEEK (PBSP) through the mediated action of polydopamine (Fig. 6 (d)). When PBSP is stimulated by LIPUS, it can generate stable electrical energy and regulate Piezo1-induced Ca^2+^ influx and Akt/GSK3β/β-catenin pathway.

The advantages of the ultrasonic response strategy are its non-invasiveness and good tissue penetration depth, and LIPUS itself has a safe clinical application basis, which is easy to cooperate with piezoelectric materials to enhance the osteogenic effect. Despite this, there are still limitations of this technique in practical applications. Ultrasound energy is prone to uneven distribution, reflection and attenuation in complex and porous organoids, which affects the uniformity and repeatability of the stimulus. Inappropriate parameter settings may trigger unexpected thermal effects and cause damage to cells. In addition, the quantitative analysis of the coupling efficiency of ultrasound and materials and the deep molecular mechanism are still the core topics that need to be continuously explored in this field.

#### 4.1.4 Other responsive materials

Light and heat as other main physical stimuli are also commonly used in the dynamic culture environment of bone tissue engineering. Light and heat responsive materials can accurately regulate their stiffness and macroscopic morphology by external light sources or temperature changes, so as to dynamically simulate the microenvironment evolution during bone development. These materials can regulate the physical properties such as surface topology and elastic modulus in real time through photothermal response, which directly affects the adhesion, extension and osteogenic differentiation process of stem cells. In addition, responsive materials based on photothermal effect can also realize the on-demand release of growth factors and the dynamic construction of cellular microenvironment, which provides a more accurate means of spatiotemporal regulation for the study of the dynamic evolution of bone organoids under specific physiological or pathological conditions (see Table 4).

### 4.2 Comparation of response strategies in bone organoid

Although the above intelligent responsive materials have shown significant potential in simulating dynamic bone microenvironment, in the practical application of constructing functional bone organoids, various strategies are still limited by specific physical and biological bottlenecks, and no universal solution has been formed. First, there is an irreconcilable contradiction between tissue penetration depth of physical stimuli and spatiotemporal resolution. As an entity with a specific volume and a highly mineralized matrix, the precise regulation of deep cells in bone organoids faces severe challenges. The magnetic response strategy can penetrate the deep mineralized matrix without attenuation, which is extremely suitable for the construction of large-scale organoid models or *in vivo* implants [137]. However, it is difficult for magnetic fields to construct steep gradients on the micrometer scale, which limits their ability to differentially regulate specific microregions, such as the organoid core hypoxic region [130]. In contrast, although photothermal or photodynamic strategies have extremely high spatial and temporal resolution, they are limited by the tissue penetration depth of light (usually <2 mm for visible light and <1 cm for near-infrared light), and are often only suitable for thin-layer microfluidic chip models, which are difficult to meet the needs of macroscopic bone regeneration scaffolds [138]. Although the ultrasound strategy is superior to the light control technology in terms of penetration, the sound wave is easy to reflect and scatter in the porous scaffold. The resulting stationary wave effect may lead to local stress concentration or thermal damage, and the uneven distribution of energy in the complex porous structure also affects the repeatability of stimulation [139]. Secondly, from the perspective of bionic logic, the passive response and active intervention modes represented by different materials have their own advantages and disadvantages. The passive response strategy represented by piezoelectric materials relies on the movement and deformation of bone itself to generate endogenous electrical signals. This self-powered mode is highly consistent with the stress-potential feedback mechanism of natural bone and is suitable for simulating long-term steady state maintenance in physiological states [124], [125]. However, its efficacy is completely limited by the amplitude of mechanical load, and it is difficult to effectively activate the piezoelectric effect for bedridden patients who lack exercise capacity or organoids in the early stage of static culture. In contrast, magnetic and ultrasonic responsive materials are active intervention strategies that allow researchers to precisely program the frequency, intensity, and duration of stimuli through external devices [137], [139]. This controllability makes it more suitable as a powerful induction method for precise intervention during critical windows of organoid development, such as the early stage of vascularization, but faces the problem of continuous dependence on external devices in long-term implantation. To more clearly guide the selection of materials in the construction of bone organoids, we systematically compare the above strategies in terms of energy source, tissue penetration, and spatiotemporal resolution in Table 5. In short, piezoelectric materials, while providing the most biomimetic electrical microenvironment, rely on continuous mechanical loading. The magnetic response strategy has the best tissue penetration depth, which is suitable for the construction of large organoids or *in vivo* implantation models. The ultrasound response strategy has advantages in clinical translation potential (see Table 6).

**Table 4:** Summary of various responsive materials and their functions.

Responsive Material	Stimulation Type	Functions	Cell type	Reference
GelMA/BTO	Piezoelectric	Enhanced cell viability and effectively guided their early osteogenic lineage commitment. With a >1.5-fold increase in bone volume fraction.	hPDLSCs	[104]
BTO-coated Titanium Alloy	Piezoelectric	Synchronously drove the osteogenic and angiogenic self-assembly processes within the engineered niche. Enhanced *in vivo* BV/TV by >30%	rBMSCs	[105]
HAP/BTO	Piezoelectric	Steered M2 macrophage polarization to establish a permissive osteoimmune microenvironment, triggered a ∼2-fold increase in VEGF expression, and synergistically facilitating coupled multicellular morphogen.	BMSCs & RAW264.7 (Mouse macrophages)	[106]
PVDF/TiO_2_	Piezoelectric	Upregulated terminal OCN to accelerate ECM mineralization. With >2-fold enhancement in matrix mineralization.	MSCs	[107]
PVDF-TrFE/BNNTs	Ultrasound	Accelerated the maturation. Increased intracellular calcium deposition and osteocalcin expression by >30%.	SaOS-2 (Human osteoblast-like cells)	[108]
PVA/PVDF/Silver	Piezoelectric	Provided sustained mechanobiological activation essential for the spatial reconstruction of the osteochondral interface.	BMSCs	[109]
PLLA	Mechanical Activation	Orchestrated spatiotemporal cues to effectively drive autonomous osteochondral tissue evolution.	Chondrocytes & MSCs	[110]
Polypyrrole	Electrical Stimulation	Translated transient electro-mechanical cues into sustained mechanotransduction and robust osteogenic biomarker production, with ∼2-fold higher ALP activity.	hMSCs	[111]
Dopamine/Fe_3_O_4_	SMF	Delivered wireless mechanotransduction to significantly enhance the autonomous biomineralization of macroscopic constructs.	MSCs	[112]
MNP/PLLA/Magnesium Oxide	SMF, 80 mT	Accelerated Mg^2+^ uptake (>40%) to synergistically trigger intracellular mineralization cascades, reinforcing cellular activity and robust osteogenic fate decisions.	BMSCs	[113], [114]
CoFe_2_O_4_/PLLA	SMF	Enhanced osteoblast proliferation (>1.5-fold) through combined mechanical and electrical stimulation.	MC3T3-E1	[115]
CoFe_2_O_4_/PVDF-TrFE	SMF	Directed osteogenic fate decisions through upregulate COL-1 and ALP gene expression by ∼1.5 to 2 folds.	rBMSCs	[116]
PLGA/MNP	SMF	Directly upregulated the critical mechanosensor Piezo1 to prime the cells for dynamic self-organization.	hBMSCs	[117]
PEG/MNP	SMF	Guided the spatiotemporally organized co-culture and coupled assembly of endothelial and osteogenic lineages.	ASCs (Adipose-derived Stem Cells)	[118]
Fe_3_O_4_/gelatin	AMF	Exerting periodic shear stress to physically drive BMSC fate decisions and macroscopic osteogenesis.	MSCs	[119]
CoFe_2_O_4_@BTO/PVDF-TrFE	AMF	Converted remote AMF signals into internal biophysical stimuli to drive robust osteo-angiogenic co-maturation.	BMSCs	[120]
PVDF	LIPUS	Acoustic-to-electric conversion exponentially amplified ALP activity, providing global cues for organoid mineralization.	MSCs & Osteoblasts	[121]
BTO/OCS/gelatin	LIPUS	Translated acoustic waves into intracellular calcium transients, activating PI3K/AKT and MEK/ERK cascades to orchestrate BMSC differentiation pathway.	BMSCs	[122]
Polydopamine/BTO-modified PEEK	LIPUS	Produced stable electrical energy under LIPUS; regulated Piezo1; induced Ca2+ influx and activated the Akt/GSK3β/β-catenin pathway.	rBMSCs & Osteoblasts	[123]

**Table 5:** Comparison of different stimulus response strategies.

Strategy	Mechanism	Tissue penetration	Spatial-temporal resolution	Advantages	Disadvantages
Electrical response	Mechanical energy to electrical energy.	Surface stimulation: around hundreds micrometer. Invasive stimulation: controllable depth.	Surface stimulation: around hundreds micrometer. Invasive stimulation: At the single-cell level.	Simulate the piezoelectricity of natural bones.	A signal can only be generated by relying on a continuous mechanical load. Some piezoelectric ceramics are non-biodegradable.
				Self-powered	
Magnetic response	Magnetic energy to mechanical energy or thermal energy	High, around several centimeters.	Medium, usually at the centimeter level.	Remote non-invasive control.	Required large-scale magnetic field generating devices.
				Can simultaneously achieve imaging and treatment.	The potential biological safety issues regarding the long-term retention of MNPs.
Ultrasound response	Sound energy to electrical energy or mechanical energy	Extremely high, reaching up to 10-15 cm or even deeper.	High, ranging from millimeter level to sub-millimeter level	Good clinical application foundation.	Sound energy is prone to attenuation and reflection in porous structures.
				Can be combined with piezoelectric materials to achieve efficient acoustic-electric conversion.	The thermal effect threshold must be strictly controlled.
Light/heat response	Light energy to thermal energy or mechanical energy	Medium, around several millimeters	High, ranging from millimeter level to sub-millimeter level	Precise control of time and space.	Limited penetration depth
				Triggered release of drugs at specific locations.

**Table 6:** Method of integrating mechanical stimulation into the bone organ-on-a-chip.

Stimulation Type	Integrated Method	Results	Cell type	Reference
Compression	Pressurize by pneumatic device. Direct compression	Excessive compression promotes the expression of osteoarthritis-related genes.	Primary chondrocytes	[146], [163], [164]
Compression	Pressurize by pneumatic device. Dynamic hydraulics.	Moderate mechanical stimulation significantly increased the expression of osteogenic genes.	hBMSCs & hADSCs	[166]
Compression	Non-contact mechanical stimulation controlled by an external magnetic field.	Non-contact mechanical stimulation regulation was achieved, significantly enhancing MSC recruitment efficiency by > 2-fold.	Chondrocytes & MSCs	[147]
Fluid Shear Stress	Microfluidic technology.	Fluid shear stress promoted cell proliferation and upregulated early osteogenic gene expression by > 1.5-fold.	MC3T3-E1	[167]
Fluid Shear Stress	Microfluidic technology.	Osteogenic differentiation markers were significantly upregulated, indicating that fluid flow could effectively induce osteocyte maturation.	hMSC-derived osteoblasts/osteocytes	[148]
Fluid Shear Stress	Microfluidic technology.	Fluid shear stress can reduce RANKL/OPG ratio in osteocytes, thereby inhibiting osteoclast differentiation.	MLO-Y4 & RAW264.7	[168]
Fluid Shear Stress	Microfluidic technology.	Mechanical response has a dual role in cancer bone metastasis.	Osteocytes & breast cancer cells	[169]

### 4.3 Degradation of smart materials and substitution of matrix

In the engineering construction logic of bone organ, the role of the scaffold should be a temporary morphogenesis template rather than a permanent implant. The ideal engineering strategy is to establish a dynamic balance between scaffold degradation and tissue regeneration. As cell self-assembly proceeds and the deposition of the own ECM occurs, the intelligent scaffold should gradually degrade and ultimately achieve a smooth transition from artificial materials to the organ-like structure. The core of this process lies in the matching of degradation kinetics. The degradation rate of the scaffold must be synchronized with the generation rate of the new tissue [140]. If the degradation is too fast, the new tissue will lose its mechanical support and collapse. If degradation is too slow, the residual material will continue to occupy a limited physical space, directly limiting the deposition and rearrangement of the nascent mineralized matrix. More importantly, when external dynamic stimulation is applied, the undegraded high-stiffness scaffold will absorb most of the mechanical energy, hindering the effective transmission of mechanical signals to cells and the new matrix, thereby inhibiting the further functional maturation of the tissue. Therefore, an ideal smart scaffold should exhibit surface erosion rather than bulk degradation to ensure that its geometric connectivity and mechanical properties decay linearly rather than abruptly during degradation. Second, for smart responsive materials, the degradation process itself can be designed as an active biological regulation. Unlike inert materials, the degradation products of smart materials tend to have specific biological activities. For example, Mg^2+^ released from magnesium-based scaffolds during degradation is not only a raw material for mineral deposition, but also stimulates the secretion of calcitonin gene-related peptide (CGRP) from nerve terminal by regulating the local microenvironment, thus promoting the coupling of angiogenesis and osteogenic differentiation [141]. This ion release can have a synergistic effect with the stimulation of an external physical field, such as a magnetic field: the physical field opens mechanosensitive channels, and the degraded ions act as cofactors to enhance downstream signaling.

However, not all degradation products of stimulus-responsive materials are biologically active. At present, high-performance piezoelectric ceramics (such as BTO) and magnetic nanoparticles (such as Fe_3_O_4_) are mostly difficult to degrade in physiological environments [142]. When bone organoids are used as disease models for drug screening, the residual nanoparticles may interfere with the evaluation of drug metabolism kinetics through non-specific adsorption. However, *in vivo* application, the long-term retention of materials may induce chronic inflammation or foreign body reaction, which is contrary to the original intention of immunoregulation. Even for degradable piezoelectric polymers (such as PLLA), their piezoelectric properties often decay rapidly with polymer degradation [143], and the mismatch between functional degradation and tissue regeneration rate may lead to the loss of necessary dynamic signal support before the new tissue maturates. Therefore, future research needs to turn to the development of new stimulus-responsive materials based on endogenous biomolecules to achieve wireless, precise, and long-term regulation of deep tissues while solving biosafety issues.

### 4.4 Challenges and perspectives in engineering dynamic microenvironments

While current stimuli-responsive materials and bioreactors have demonstrated significant efficacy in promoting osteogenesis, several critical bottlenecks must be addressed to fully transition from engineered templates to biologically autonomous bone organoids. First, native bone organogenesis requires gradient-specific biophysical cues, whereas most current stimuli-responsive systems provide global or bulk stimulation. For instance, homogeneous magnetic or acoustic fields often trigger uniform cellular responses across the entire scaffold, failing to recapitulate the complex, patterned mechanotransduction characteristic of natural embryonic development. Future engineering strategies must focus on developing programmable smart materials capable of delivering high-resolution, spatiotemporally precise signals to drive localized lineage commitment and patterned vascularization. Second, to facilitate the systematic reduction of external support, next-generation responsive materials must be intrinsically biodegradable. Developing metabolizable piezoelectric polymers or enzymatically cleavable magnetic hydrogels, whose degradation profiles strictly synchronize with the deposition rate of the endogenous ECM, represents a crucial direction for future research.

In summary, the construction of dynamic microenvironment of bone organoids should not be regarded as the superposition of single technologies, but rather a system engineering integrating mechanical engineering, materials science and cell biology. This chapter systematically describes the dual strategies of external physical field loading and in situ mediated by smart materials. The former provides precise and controllable macroscopic mechanical commands by means of bioreactors, and the latter realizes wireless and microscopic precision of physical stimulation by means of smart responsive materials. More importantly, with the gradual degradation of engineered scaffolds and matrix replacement, the main body of mechanical support will smoothly transition from artificial materials to new biological tissues. This spatiotemporal synergy of dynamic stimulation and dynamic evolution is the key to realize bone organoids from simple cell stacking to real physiological functions, and also lays a solid physical and material foundation for the integrated organ-on-a-chip platform discussed in the following chapters.

## 5 Construction of integrated bone organ-on-a-chip platform and disease model

Organ-on-a-chip technology provides a highly integrated and controllable *in vitro* platform for the study of bone organoids. The core advantage of organ-on-a-chip lies in its high degree of integration. It does not simply reduce the size of organoids into the chip, but integrates the control and observation ability of multiple dimensions such as cellular microenvironment, biomechanical stimulation, biochemical signal gradient and real-time sensing detection into a unified and controllable micro-platform. Through the principle of microfluidic, microstructures are constructed on the chip, and ECM and various cell types are embedded. This platform integrates engineered scaffolds, bone organoid models and external physical stimuli to accurately simulate the complex bone microenvironment and biological functions [11]. Such systems can not only replicate organotypic structures, but also support non-invasive and real-time monitoring of dynamic changes in cell behavior and biomarkers due to their excellent optical properties, thus making up for the limitations of traditional endpoint detection methods [144]. Therefore, organ-on-a-chip technology provides a powerful tool with more physiological relevance and controllable flux for drug screening, disease mechanism research and personalized medicine [145].

### 5.1 Integrated design principle of bone organ-on-a-chip

As an efficient integrator, bone organ-on-a-chip is designed to systematically integrate four core elements: organoids, functional scaffolds, physical stimulation modules and sensor monitoring units. Firstly, through the precise design of organoid chambers, the chip can realize the precise spatial arrangement of multiple cell types and simulate the organization and proximity of cells *in vivo*. Secondly, stimulation integration modules enable a variety of physical stimuli to be applied to the target area with precision and control. Moreover, the integration of sensing and monitoring is another major advantage of the chip, such as the integration of microelectrodes to detect bioelectrical signals or pH changes, or the use of its excellent optical properties for real-time fluorescence imaging to continuously track the cell response and microenvironment parameters. Ultimately, the combination of these capabilities sets the stage for the construction of feedback control loops that automatically adjust stimulus parameters based on real-time readings from sensors, enabling truly dynamic, adaptive physiological or pathological simulations.

### 5.2 Bone organoid construction with 3D printing

While traditional organoid derivation relies mainly on the spontaneous self-assembly of stem cells, this stochastic process fundamentally lacks the macroscopic architectural control required for load-bearing skeletal tissues. By precisely depositing cells, biomaterials, and morphogenetic factors layer-by-layer, 3D bioprinting actively imposes spatial constraints and acts as a macroscopic morphogenetic template, significantly accelerating the application of organoids in functional tissue regeneration [151].

At the core of this technology is the rational design of bioinks, which function not merely as passive structural fillers, but as an active, dynamic microenvironment that programs cell fate and self-assembling. Recent advancements have shifted towards hybrid and dynamic bioinks to better recapitulate the native bone niche. For instance, the incorporation of bone matrix-inspired inorganic components, such as hydroxyapatite (HA), into hybrid bioinks can proactively drive the continuous self-mineralization of large-scale bone organoids [7]. Furthermore, by tailoring the physicochemical properties of matrix-mimicking bioinks, researchers can empower MSCs to spontaneously self-organize into fully differentiated, vascularized bone microarchitectures, successfully recapitulating autonomous developmental trajectories even in ectopic environments [152]. Utilizing DNA-encoded dynamic hydrogels during the 3D bioprinting process can provide dynamic mechanical support and precise cell-adhesion sites tailored for chondrogenic organoids [153]. Similarly, intelligently designed hydrogels can act as sustained-release systems, providing a continuous supply of biochemical signals to accelerate the maturation of engineered organoids [154]. Leveraging these advanced bioinks, researchers have successfully printed prevascularized bone organoids capable of rapid in situ cranial bone reconstruction [149]. Despite the ability of 3D bioprinting to construct complex cellular matrices, the dense, mineralized nature of bone organoids inherently restricts nutrient diffusion. To address this bottleneck, advanced bioprinting strategies—such as utilizing sacrificial bioinks or coaxial extrusion—are commonly integrated to architect perfusable internal micro-channels, thereby facilitating subsequent vascular network anastomosis (detailed engineering solutions for vascularization are thoroughly discussed in Section 5.3).

At the same time, the development of 4D bioprinting technology provides a physical means for constructing a dynamic responsive microenvironment. Different from static 3D printing, 4D printing introduces time as the fourth dimension, and uses the response characteristics of smart materials to environmental stimuli to produce smart scaffolds that can autonomically change shape or function during culture [155]. Such time-evolving scaffolds are able to mimic movements during bone development or release bioactive factors at specific time points, thereby enabling dynamic guidance of cell fate [156].

### 5.3 Construction and functionalization of bone organ-on-a-chip

Organ-on-a-chip technology provides a highly controllable and biomimetic *in vitro* platform for studying the role of mechanical stimulation in bone biology. It can systematically integrate bone organoids, engineered matrices, and a variety of mechanical loading methods to achieve accurate simulation and real-time analysis of mechanical signal transduction processes in complex bone microenvironment.

#### 5.3.1 Engineering solutions for vascularization within chips

**Fig. 7:**
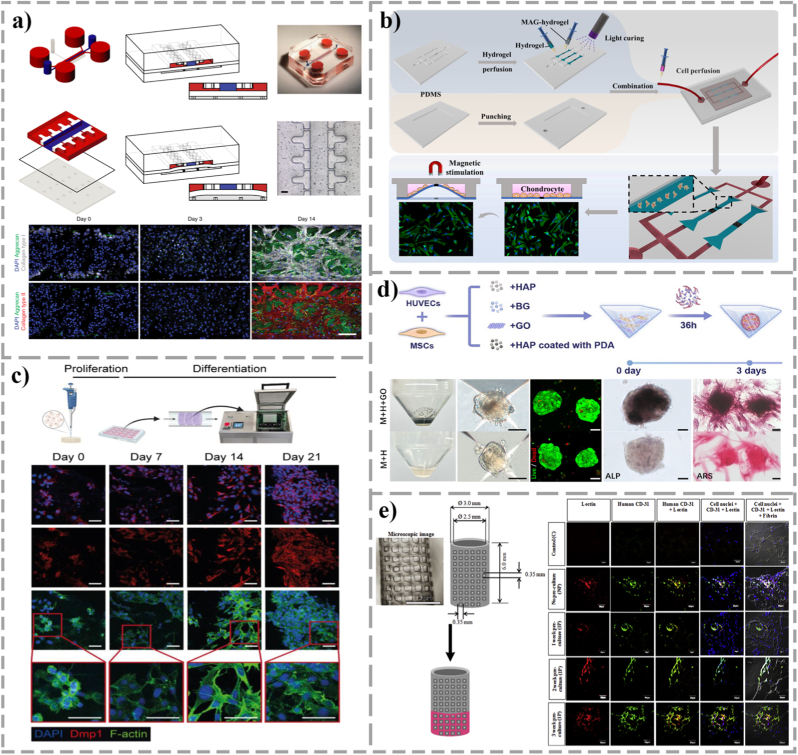
Culture platform design that integrates engineered scaffolds and external mechanical stimulation and culture of bone organoids. (a) Combining a pneumatic device with a chip to apply dynamic compression on chondrocytes provides an important model for the study of the mechanism of osteoarthritis. Scale bar: 100 μm. (Adapted with permission from Ref. [146]. Copyright 2019, Springer Nature.) (b) A biomimetic cartilage organ-on-a-chip system integrating magnetically responsive hydrogel achieves non-contact mechanical stimulation regulation through external magnetic fields, demonstrating the application of engineered mechanical loading strategies in the construction of bone organoids and mechanical transduction at bone-implant interfaces. (Adapted from Ref. [147] under a CC BY 4.0 license.) (c) Through microfluidic technology, different magnitudes of fluid shear forces were applied to the 3D scaffold implanted with IDG-SW3 osteoblasts to accurately control the mechanical microenvironment of cell culture. Scale bar: 50 μm. (Adapted from Ref. [148] under a CC BY 4.0 license.) (d) Bone organoids with pre-vascularized characteristics were constructed by integrating MSCs, endothelial cells and graphene oxide particles. Scale bars: 50 μm. (Adapted with permission from Ref. [149]. Copyright 2025, John Wiley and Sons.) (e) hMSCs and HUVECs were co-cultured in a PPF/fibrin scaffold to successfully realize the culture and vascularization of bone organoid *in vitro*. Scale bars: 50 μm. (Adapted with permission from Ref. [150]. Copyright 2016, Elsevier.)

The establishment of functional vascular network is a key link to maintain the survival of large bone organoids, promote their functional maturation, and achieve efficient transport of nutrients and signaling molecules [17], [157]. With its precise fluid control and spatial design capabilities, chip technology provides an ideal platform for the construction of highly bionic vascularized bone organoids. Current strategies mainly rely on the combination of cell co-culture with engineered biomaterials. For example, Duan et al. [149] successfully constructed bone organoids with prevascularized characteristics by co-culturing MSCs, endothelial cells and graphene oxide (GO) particles in the chip, achieved the rapid construction of mature bone organoids (Fig. 7 (d)). Chiesa et al. [158] adopted a sequential seeding strategy, in which hMSCs were first seeded on the gelatin-HA scaffold, and then introduced into human umbilical vein endothelial cells (HUVECs) into the macropores to form an organoid model with a rich vascular network within 4 weeks. In addition, multi-material bioprinting technology has also been used to fabricate vascularized bone organoids. Mishra et al. [150] used a highly rigid PPF printed framework to provide the mechanical boundary conditions required for bone formation, while accurately filling Fibrin hydrogel containing HUVECs in specific gaps of the framework. This spatially resolved soft and hard alternating design forces endothelial cells to stretch and tube along preset soft tracks to form microcapillaries in dense tissues (Fig. 7 (e)). However, engineered bone organoids face a unique morphogenetic challenge: the continuous deposition of a dense, mineralized matrix severely restricts internal mass transfer and physically obstructs the long-term maintenance of these nascent vessels. To overcome this biophysical bottleneck, advanced engineering interventions are imperative to guide macro-vascular morphogenesis. Coaxial 3D bioprinting combined with sacrificial template strategy has emerged as an engineering solution, enabling the simultaneous extrusion of a tissue-laden bioink (via the outer nozzle) and an endothelial cell-laden sacrificial ink (via the inner nozzle). During the fabrication phase, the sacrificial ink provides essential mechanical support for the fluidic architecture, while upon physiological culture, it actively dissolves to yield an interconnected, open channel network. Concurrently, the encapsulated endothelial cells are liberated to adhere and spread along the luminal walls, effectively transforming bare physical conduits into a biologically functionalized vascular network. Leveraging this paradigm, researchers [159] have successfully achieved the fabrication and prolonged *in vitro* cultivation (≥20 days) of macroscopic, vascularized tissue constructs exceeding 1 cm in dimensions. Furthermore, vascular morphogenesis is a dynamic, continuous process. In extended culture periods (typically >4 weeks), the nascent vascular network faces a profound risk of regression due to the depletion of continuous trophic support and the absence of physiological hemodynamic forces [160]. Therefore, to prevent structural collapse and sustain long-term vascular stability, these morphogenetic templates must be dynamically integrated with macroscopic perfusion bioreactors or microfluidic organ-on-a-chip platforms [161], [162].

#### 5.3.2 Physically-stimulated organoid formation

Integrating controllable physical stimuli on the chip platform is the core strategy for guiding bone-like organs to achieve structural maturity and functional differentiation. Compression and stretching stimuli are widely used to simulate the mechanical load of bones. For instance, a culture system constructed based on the deformable property of polydimethylsiloxane (PDMS) can apply controllable pressure to cells through an air-driven device in vertical, horizontal, or vortex directions [163]. For example, deforming the PDMS membrane by applying pressure to the bottom chamber enables vertical compression of the culture matrix containing chondrocytes and PEG-hydrogel in the upper layer, and this stimulation induces a shift from a homeostatic to a catabolic and inflammatory phenotype and promotes the expression of osteoarthritis-related genes [146], [164]. This type of mechanical loading strategy has also been used to construct osteochondral organoid models to simulate the physiological conditions at the joint more economically by combining mechanical stimulation with specific media [165]. In research applications, Park et al. [166] combined pneumatic devices with organ-on-a-chip to apply dynamic hydraulic compression of 1 psi or 5 psi to hMSCs and hASCs and showed that mechanical stimulation significantly increased early osteogenic lineage commitment. Moreover, hMSCs showed higher mechanical sensitivity and osteogenic differentiation efficiency than hASCs. Lee et al. [164], on the other side, designed a two-chamber organ-on-a-chip that used pressure changes to drive PDMS membranes to compress chondrocytes in alginate hydrogel to simulate physiological compressive stress. On this basis, Occhetta's team [146] systematically studied the effect of compressive stress on articular cartilage, and found that 10% physiological compression could enhance the expression of cartilage markers such as collagen type II, while 30% hyper-physiological compression induced the up-regulation of matrix degrading enzyme MMP13 and inflammatory factors IL-6 and IL-8. This precise mechanical mapping demonstrates how varying strain magnitudes can either reinforce the cartilaginous morphogenetic template or divert the developmental trajectory toward a pathological phenotype, providing a crucial *in vitro* model for osteoarthritis mechanopathology (Fig. 7 (a)).

As the main mechanical stimulus for osteocytes *in vivo*, the role of fluid shear stress has been deeply explored on chip. In the study by Rindt et al. [148], IDG-SW3 osteoblasts were cultured in a poly (L-lentate-cotrimethylene carbonate) (PLTMC) 3D microporous scaffold and significantly upregulated osteogenic differentiation markers such as ALP, DMP-1 and SOST under fluid shear at a flow rate of 1.7 mL/min. This demonstrates that convective fluid flow acts as a potent physical cue to orchestrate the late-stage maturation of osteocytes, highlighting the necessity of integrating hydrodynamic boundary conditions in engineered morphogenetic templates (Fig. 6 (c)). The precision of flow control is critical to the application of shear stress, for example, the use of a syringe pump or a pressure control system can generate shear forces of a specific magnitude within a microflow channel. One study showed that under 0.5 dyn/cm^2^ (corresponding to 50 μL/min) shear stress, the proliferation of MC3T3-E1 in collagen matrix increased 2.4-fold, while under 0.3 dyn/cm^2^ (30 μL/min), the proliferation increase was not significant but ALP activity increased 1.6-fold. This indicates that different levels of shear stress can reprogramming the developmental trajectory by regulating nutrient transport and cell metabolism [167]. In addition, the multi-channel microfluidic device developed by Middleton et al. [168] shows the application of 1 Pa fluid shear stress can reduce the RANKL/OPG ratio in osteocytes, thereby inhibiting osteoclast differentiation, while unstimulated osteocytes can increase RANKL expression and decrease OPG expression due to increased apoptosis, promoting osteogenesis. These results revealed cross-channel communication between osteocytes and osteoclast precursor cells in response to mechanical stimulation. Microfluid platform not only provides support for the study of disease mechanisms such as osteoporosis and bone metastasis, but also further explores the role of mechanical stimulation in cancer metastasis. Studies [169] have shown that MLO-Y4 osteocytes can inhibit extravasation and migration of breast cancer cells by increasing ATP release, decreasing RANKL secretion and decreasing IL-6 expression under fluid shear at 1 Pa and 1 Hz. At the same time, mechanical stimulation also up-regulates factors such as PGE-2 and TGF-β1, which may promote tumor metastasis by enhancing MMP expression, indicating that mechanical response has a dual role in cancer bone metastasis.

In addition, electromagnetic fields are also successfully compatible with the chip platform as non-direct contact physical stimuli devices. Liu et al. [147] developed a biomimetic cartilage organ-on-a-chip system integrated with a magnetically responsive hydrogel to achieve non-contact regulation of mechanical stimulation through an external magnetic field (Fig. 7 (b)). Aldebs et al. [135] designed a synergistic system of pulsed EMF with superparamagnetic iron oxide nanoparticles (SPIONs) to significantly induce the early differentiation of human ASCs into the osteoblast lineage. This pulsed EMF emitted a 1 mT signal at a pulse frequency of 15 Hz and was repeated 20 times, which was in the extremely low frequency range. This wireless innovation effectively translates remote physical signals into deep-tissue mechanotransduction cascades, establishing a dynamic microenvironment to orchestrate early cellular self-organization without direct physical contact.

#### 5.3.3 Multi-tissue interface and multi-organ crosstalk model

Bone does not exist in isolation in physiological state, but maintains systemic homeostasis through complex communication with cartilage, nerves and even distal organs. The unique advantage of chip platform lies in its ability to reconstruct these complex interactions. At present, three types of representative models have been derived: tissue interface model, innervation model, and multi-organ-on-a-chip. Tissue interface models focus on simulating interactions between anatomically closely contiguous tissues. For example, Lin et al. [170] successfully constructed an osteochondral organ-on-a-chip model by inducing region-specific differentiation of iPSC in a unified hydrogel matrix, which provides a platform for studying mechanical signal transduction and material exchange at the joint interface. Innervation models were used to reveal the fine regulation of bone metabolism by the nervous system. Using a microfluidic device with microgroove structure to co-culture dorsal root ganglion neurons and MSCs, studies have confirmed that neurite extension can not only enhance the metabolic activity of MSCs, but also significantly promote the osteogenic differentiation process by up-regulating the expression of key osteogenic genes [171]. Multi-organ-on-a-chip hydrodynamically connect organoids from different tissues through microfluidic circulatory systems to simulate systemic interactions at the organism-level. The chip system including heart, liver, bone, skin and other organs developed by Kacey et al. [172] is a typical representative of this direction. However, the synovial-cartilage co-culture model constructed by Rothbauer et al. [173] successfully simulated the pathological crosstalk between tissues in the process of arthritis.

### 5.4 Construction and application of pathological models on bone organ-on-a-chip

The ultimate application of engineered bone organoids extends beyond modeling healthy development as it provides a platform for deconstructing the spatiotemporal progression of skeletal pathologies. By integrating tunable morphogenetic templates with microfluidic organ-on-a-chip systems, researchers can precisely replicate the pathological mechanobiological niches of various bone diseases, offering critical comparative advantages over traditional *in vitro* models.

#### 5.4.1 Degenerative and metabolic bone disease models

Chronic bone diseases, such as osteoarthritis (OA) and osteoporosis (OP), are fundamentally driven by mechanical wear and metabolic decoupling. Traditional 2D cultures completely fail to capture the load-bearing nature and multi-tissue boundary conditions of articular joints. In contrast, engineered organoid-on-a-chip systems can systematically decouple these complex variables. For OA modeling, Dönges et al. [174] engineered hMSC-derived cartilage organoids that accurately recapitulated the key pathological features of OA under specific environmental constraints. Extending this to multi-tissue crosstalk, synovial organoids developed by Sun et al. [175] elucidated the regulatory miR-138/FOXC1/HIF signaling axis, proving the indispensable role of synovial-chondrocyte communication in alleviating metabolic imbalances. Furthermore, Limraksasin et al. [165] utilized mouse iPSCs to construct 3D osteochondral organoids, successfully simulating the synergistic pathological trajectory of cartilage degeneration coupled with subchondral bone sclerosis. For OP models, the focus shifts to the dynamic imbalance between osteogenesis and osteoclastogenesis. Bukhari et al. [176] constructed humanized 3D vascularized bone organoids to mimic the rapid loss of mineralized matrix induced by estrogen deficiency. Similarly, Iordachescu's group [14] and Liu et al. [177] established 3D humanized trabecular organoids that dynamically modeled the destruction of bone metabolic homeostasis. The superiority of these engineered templates lies in their ability to sustain the long-term, coupled self-assembly of osteoblasts and osteoclasts, a feat impossible in standard 2D plastic dishes where dynamic bone remodeling cannot occur.

#### 5.4.2 Bone defect and regeneration models

Traditional defect models rely heavily on *in vivo* animal surgeries, which are plagued by species-specific differences that mask human cellular dynamics. Conversely, engineered morphogenetic templates allow researchers to seamlessly recapitulate the temporal developmental trajectory of human fracture repair *in vitro*. By combining 3D bioprinting with smart biomaterials, researchers have constructed multi-organ-on-a-chip that simulate the dynamic progression of hematoma formation, cartilaginous template deposition, and subsequent biomineralization [178]. Notably, Nilsson et al. [179] utilized human periosteum-derived cells to fabricate callus organoids, successively replicating the classic morphogenetic sequence of endochondral ossification—from chondrocyte hypertrophy to the cartilage-to-bone transition. Ouyang et al. [178] further scaled this concept, employing digital light processing (DLP)-based bioprinting to mass-produce BMSC-encapsulated callus organoids, providing a highly standardized platform for regenerative screening.

#### 5.4.3 Bone tumor and infection models

In the context of bone malignancies (e.g., osteosarcoma) and metastatic cascades, the superiority of engineered morphogenetic templates over simple 3D gels or rigid 2D cultures is most profoundly demonstrated through their integration of mechanobiology. During tumor progression, metastatic cells actively remodel their surrounding microenvironment, inducing a pathological mechanical stiffening of the ECM through excessive collagen crosslinking. This tumor-induced matrix stiffening, in turn, amplifies intracellular mechanotransduction pathways, creating a positive feedback loop that accelerates aggressive tumor invasion. Traditional 2D models and homogenous models cannot capture this dynamic reciprocity.

By utilizing 3D engineered templates with tunable stiffness gradients, researchers can systematically decouple these physical variables. These gradient templates uniquely elucidate how varying substrate moduli actively dictate tumor extravasation, migration speed, and metastatic colonization—mechanisms that are completely obscured on infinitely rigid 2D petri dishes [180], [181]. For instance, He et al. [182] successfully established patient-derived osteosarcoma organoids that retained the histological features and tumor-infiltrating lymphocytes of the primary tumor. Furthermore, Han et al. [183] used 3D printing to construct a highly bionic bone microenvironment model and realized long-term culture of cells derived from breast cancer patients with bone metastasis. The ability to program the initial mechanical stiffness of these scaffolds allows researchers to directly observe how engineered physical constraints inhibit or promote tumor progression. Additionally, for infectious pathologies, the osteomyelitis model simulates bone infection by introducing pathogenic microorganisms into the engineered osteoimmune niche. You et al. [184] developed bone cement composites with both osteogenic and antibacterial functions. For drug delivery, injectable naringin loaded hydrogel microspheres [185] and bone-targeted mesoporous silica nanoparticles [186] have shown synergistic potential for anti-infection and osteopromotion.

Pathological models chip offer unparalleled advantages in regulating microenvironmental factors (especially mechanical stiffness), executing spatiotemporal analyses, and preserving patient-specific heterogeneity, establishing them as formidable platforms for pharmacological screening. However, pushing these models toward clinical translation presents distinct bioengineering challenges. Degenerative models require long-term structural stability to capture slow-progressing pathologies. Regeneration models struggle to integrate the highly dynamic biomechanical loading regimens required for various defect topologies. Most critically, tumor and infection models face the immense challenge of spatiotemporally maintaining engineered stiffness gradients against the ECM remodeling driven by aggressive cancer cells or bacterial biofilms. Overcoming these dynamic mechanobiological hurdles remains the defining frontier for next-generation bone disease modeling.

## 6 Current challenges and future prospects

Although the integration of engineering strategies such as biomimetic scaffolds and dynamic microenvironmental stimulation have significantly advanced the derivation and maturation of bone organoids, the field is still in its infancy. However, to make the leap from laboratory prototypes to clinical-grade products, the field still needs to overcome biologic challenges such as insufficient vascularization, lack of standardization, and limited integration of multiple systems. To overcome these hurdles, future research must shift from isolated technical improvements to systematic paradigm shifts, focusing on the deep convergence of multidisciplinary technologies, the development of off-the-shelf translational products, and the establishment of robust regulatory frameworks.

### 6.1 Deep technology convergence for next generation bone organoid

As discussed in previous sections, isolated technologies—whether standalone 3D bioprinting, simple stimuli-responsive composite materials, or conventional static culture systems—are insufficient to recapitulate the highly complex, spatially restricted, and temporally dynamic niches of bone development. The next generation of engineered bone organoids mandates a deep technology convergence strategy, evolving into a highly integrated systems engineering framework. For example, embedding 3D printed morphogenetic templates directly within microfluidic organ-on-a-chip platforms, coupled with stimuli-responsive smart materials and externally controlled physical fields. In this integrated framework, 3D bioprinting first provides the precise macroscopic spatial architecture and initial cellular compartmentalization, and the microfluidic chips mimic the physiological boundaries and interstitial fluid flow of native bone. Furthermore, by doping stimuli-responsive nanomaterials into the bioinks during the printing phase, the resulting construct gains dynamic responsiveness. The external physical stimulus modules such as programmable magnetic field generators or fluid shear stress controllers integrated into the organ-on-a-chip system can then precisely and non-invasively activate these embedded smart materials. This synergy allows for the spatiotemporal delivery of mechanical or electrical signals directly to the cellular microenvironment, continuously guiding the self-organization and developmental trajectories of the organoids over long-term culture. Ultimately, this deep integration breaks the boundaries between structural engineering, material science, and microfluidics, providing a multi-scale platform to robustly replicate human skeletal embryogenesis.

### 6.2 Clinical translation of bone organoid

Although engineered bone organoids have demonstrated immense potential in *vitro* developmental and disease modeling, their ultimate objective is to address specific clinical challenges. In the realm of regenerative medicine, current strategies for bone repair (e.g. autologous bone grafting or inert prostheses) are frequently hampered by donor-site morbidity or long-term stress shielding effects. Engineered bone organoids offer a superior biological alternative capable of actively guiding the substantial regeneration of massive bone defects. Simultaneously, in the field of drug development, traditional 2D cell cultures and animal models often fail to accurately replicate the complex human skeletal microenvironment, contributing to high failure rates in clinical trials. To fully realize their potential across both domains, the manufacturing paradigm of bone organoids must transition to standardized, off-the-shelf commercial products.

To meet the demands of the pharmaceutical industry, standardized *in vitro* tools are rapidly being commercialized. Driven by global regulatory shifts, such as the US FDA Modernization Act 2.0 which encourages the transition from animal testing toward microphysiological systems, plug-and-play commercial organ-on-a-chip products have begun to emerge. For example, the Fluidic 261 chip developed by the Microfluidics Innovation Center represents a ready-to-use platform explicitly designed to match the geometry of osteon. Integrated with automated fluidic circulation and anti-clogging mechanisms, these commercial chips enable bone organoids to be cultured in a highly controlled, dynamic fluidic environment for extended periods. Such off-the-shelf microfluidic platforms significantly lower the operational barriers for biological researchers, empowering pharmaceutical companies to conduct high-throughput, reproducible disease modeling (e.g., osteoarthritis or osteoporosis) and drug screening for bone-metastatic tumors, thereby reducing the reliance on animal models. Parallel to pharmaceutical applications, the development of off-the-shelf bone organoid for tissue-engineered implantation addresses the urgent need for acute therapeutic interventions in regenerative medicine. Traditional autologous stem cell derivation entails lengthy cultivation cycles that inevitably fail to meet the narrow therapeutic windows of patients with severe bone trauma. Consequently, next-generation translational strategies are shifting towards the establishment of comprehensive “bone organoid banks”. This approach relies on utilizing universal cell sources, such as iPSCs genetically edited to knock out human leukocyte antigen (HLA) genes, thereby evading host immune rejection. By combining these hypoimmunogenic universal cells with decellularized ECM and leveraging 3D bioprinting for scalable production, batch-to-batch consistency could be enhanced. Crucially, when coupled with advanced cryopreservation technologies, these industrially manufactured organoids could be banked long-term. Upon clinical demand, surgeons can directly retrieve, thaw, and implant these ready-to-use organoids, fundamentally eliminating the critical time lag associated with personalized cell expansion and charting a pathway for large-scale clinical application.

### 6.3 Limitations and breakthroughs of current engineering strategies

#### 6.3.1 Manufacturing bottlenecks of complex topologies

Although biomimetic topological structures, such as the TPMS structure, theoretically possess excellent permeability and mechanical properties, their manufacturing remains a significant engineering challenge. However, current 3D bioprinting technologies, whether stereolithography or extrusion, often struggle to precisely replicate complex structures. Practical limitations include the relatively low resolution of the printing ink and the difficulty in removing unreacted biological ink residues from the highly convoluted porous networks. Overcoming these manufacturing bottlenecks requires the development of higher-resolution printing technologies and the collaborative advancement of new self-supporting material formulations.

#### 6.3.2 Biological coupling and functional anastomosis in vascularization

Current vascularization strategies in bone organoid engineering primarily focus on the physical co-culture of endothelial cells or the printing of sacrificial micro-channels. However, these approaches often fall short of replicating the deep biological coupling of angiogenesis and osteogenesis. In native bone development, specific vascular subsets, notably Type H vessels (characterized by high expression of CD31 and Endomucin), play a central role in orchestrating osteoblast lineage progression and matrix mineralization. Future engineering strategies must explicitly focus on spatiotemporally delivering signals (e.g., via smart biomaterials) to induce these specific developmental landmarks. Furthermore, a critical hurdle for therapeutic bone organoids is achieving “functional anastomosis”. Beyond merely sustaining endothelial networks *in vitro*, engineered organoids must be capable of rapidly integrating and forming functional anastomoses with the host vascular bed post-implantation to prevent thrombosis and core necrosis.

To overcome these biological barriers, future engineering paradigms must transition from passive structural mimicry to active, spatiotemporal microenvironmental programming. For the targeted induction of Type H vessels, programmable biomaterials—such as degradation-dependent microcapsules or stimuli-responsive hydrogels—should be engineered for the sequential and localized release of specific neuro-angiogenic coupling factors (e.g., PDGF-BB, Slit3, or HIF-1α stabilizers) that actively drive the CD31 endothelial phenotype. Additionally, integrating biomimetic fluidic shear stress via advanced microfluidic chips can mechanically precondition endothelial cells toward a bone-specific vascular lineage *in vitro* [187], [188]. To conquer the bottleneck of functional anastomosis post-implantation, researchers should synergize high-resolution sacrificial bioprinting with gradient bio-manufacturing. By spatially creating chemokine gradients (e.g., VEGF and Angiopoietin-1) within the organoid's periphery, the construct can actively recruit host endothelial progenitors to invade and connect with the pre-vascularized network. Ultimately, designing standardized “micro-anastomosis interfaces” at the boundaries of 3D-printed organoids to allowing for direct surgical suturing or rapid microfluidic docking with host arteries/veins would ensure immediate blood perfusion upon implantation, thereby paving the way for the clinical survival of large-scale bone organoids.

#### 6.3.3 Deep integration of artificial intelligence

In recent years, beyond conventional bionic scaffold design approaches, artificial intelligence (AI) technology has been deeply integrated into the design workflow of bone scaffolds, facilitating a paradigm shift from traditional parametric forward design to performance-driven inverse design. To clarify this paradigm shift, it is essential to distinguish between traditional “topology optimization” and emerging AI-based “generative design”. Traditional topology optimization is primarily a subtractive mathematical approach, which iteratively removes material from a predefined spatial boundary to meet specific mechanical constraints. In stark contrast, AI-driven generative design utilizes deep learning algorithms to learn from massive biological and mechanical datasets, enabling the autonomous, additive generation of entirely novel and highly complex structural topologies from scratch. For periodic structures such as TPMS, machine learning (ML) has been demonstrated to effectively establish nonlinear mappings between structural morphology and mechanical properties [189]. For example, Ibrahimi et al. [190] developed a hybrid feature selection model based on nonlinear regression, which enables the precise back-inference of TPMS design parameters from target modulus and permeability, thereby realizing the customized fabrication of hydroxyapatite scaffolds. To further recapitulate the heterogeneous properties of natural bone, Viet et al. [191] proposed a novel deep artificial neural network (DANN) framework specifically tailored for optimizing nonlinear functionally graded titanium alloy scaffolds. By intelligently tuning the gradient index, this framework significantly enhances yield strength while ensuring the scaffold's stiffness matches that of the host bone. In addition to periodic structures, breakthroughs have also been achieved in the research of non-periodic random structures with greater biomimetic potential. Wang et al. [192] focused on Spinodoid structures and constructed a closed-loop inverse design system by integrating backpropagation (BP) neural networks with genetic algorithms (GA), successfully resolving the challenge of achieving high matching between the anisotropic mechanical properties of scaffolds and natural bone along three orthogonal directions. In the more complex domain of topology generation, the application of generative AI is evolving from generative adversarial networks (GANs) to diffusion models. The TriTopo-LGDM model proposed by Zhang et al. [193] innovatively combines latent graph diffusion models with topological optimization priors. By incorporating multi-physics field constraints, this model enables the generation of trabecular bone scaffolds with both high geometric fidelity and mechanical adaptability, offering a novel design paradigm for personalized bone defect repair. Collectively, these studies indicate that AI-driven design strategies are progressively overcoming the bottlenecks of mechanical mismatch and structural monotony inherent in traditional methods.

However, despite these remarkable achievements in structural and geometric generation, the current application of AI in bone organoid engineering remains highly unbalanced and is severely insufficient in several critical non-structural domains. Specifically, the application of AI in multi-material compositional design optimization is largely unexplored. Natural bone is a highly complex composite, yet current AI models rarely predict the optimal combinatorial ratios of organic hydrogels, inorganic nanoparticles, and bioactive factors required to dynamically drive specific osteogenic differentiation trajectories. Furthermore, there is a profound lack of AI-driven predictive models for intelligent material degradation kinetics. Matching the degradation rate of smart biomaterials with the deposition speed of newly formed bone matrix is crucial for successful tissue integration, yet predicting this dynamic degradation balance under complex *in vivo* or microfluidic fluidic conditions remains a significant bottleneck. To overcome these deficiencies, future research must establish large-scale, standardized biomaterial databases. By coupling high-throughput screening data with multi-modal machine learning algorithms, researchers can train predictive models to map the complex nonlinear relationships between multi-material formulations, *in vivo* degradation rates, and specific osteogenic differentiation trajectories, thereby enabling AI to autonomously recommend the optimal bioink composition.

Solving the initial material and structural design bottlenecks is only the first step. Because organoid maturation is a long-term, dynamic evolutionary process, AI frameworks must be extended across the entire cultivation lifecycle. To truly revolutionize the field and overcome these bottlenecks, the future construction of bone organoids will transcend single-discipline efforts, representing a deep convergence of cutting-edge technologies. During the long-term cultivation of microphysiological systems, ML algorithms could be used to continuously mine the massive multi-dimensional data collected by biosensors and machine vision. By identifying real-time changes in cell phenotypic and metabolic characteristics, AI can achieve a closed-loop feedback mechanism to dynamically optimize cultivation conditions—such as autonomously adjusting fluidic perfusion rates or nutrient ratios to prevent core necrosis. This data-driven strategy will significantly reduce research and development costs while improving the predictive accuracy of the models. Finally, the combination of organoid technology and robotics is imperative to eliminate human error caused by manual operation. The integration of automated pipetting workstation and high-throughput microfluidic platform will realize the large-scale culture, online detection and intelligent sorting of bone organoids, which is a key step to promote the industrial application of bone organoids [194].

### 6.4 Standardization, regulation, and animal testing alternatives

As engineered microphysiological systems become increasingly sophisticated, single-tissue bone organoids are rapidly evolving into multi-organ-on-a-chip platforms that integrate the skeletal system with the immune system, liver metabolism, or tumor microenvironments. Traditionally, preclinical bone toxicology assessments and drug efficacy evaluations have heavily relied on animal models, which frequently fail to accurately recapitulate human-specific bone metabolic dynamics. By accurately modeling human physiology, these systems are poised to dramatically accelerate new drug research and development (R&D), significantly reduce clinical trial failure rates, and spearhead the transition towards animal-free testing, thereby achieving huge commercial value.

Nevertheless, to fully translate this immense commercial and clinical potential into widespread industrial application, the field must overcome severe challenges regarding standardization and ethical regulation. At present, different laboratories have great differences in cell sources (iPSCs, MSCs or primary cells), bioink formulations, and culture processes, resulting in a lack of reproducibility and comparability of research results. In order to solve this problem, there is an urgent need to establish industry standards that cover the whole chain. This not only includes setting strict quality control indicators for the purity and differentiation potential of seed cells, but also needs to develop unified physicochemical properties of biomaterials. More importantly, a standardized functional evaluation system based on microfluidic chips should be established to clarify the quantitative acceptance criteria of bone organoids in terms of mineralization, vascularization density and drug responsiveness [195], [196].

Furthermore, the highly personalized nature of bone organoids introduces complex ethical and compliance boundaries. While patient-specific organoids offer unprecedented opportunities for personalized disease modeling, they simultaneously spark intense ethical debates. Defining the legal boundaries for the *in vitro* utilization of human genetic resources, mitigating the potential off-target risks associated with gene editing, and rigorously protecting sensitive patient data are critical prerequisites. Establishing a transparent, robust, and universally accepted regulatory and ethical framework is essential to ensure the safe and sustainable commercialization of next-generation bone organoid technologies [197], [198].

### 6.5 Conclusion

In summary, the emergence of bone organoids marks a profound paradigm shift in regenerative medicine and disease modeling, fundamentally distinguishing itself from traditional BTE. While conventional BTE primarily focuses on providing passive physical support for macroscopic defect repair, bone organoids strive to recapitulate the autonomous developmental trajectories, dynamic intercellular crosstalk, and spontaneous structural self-organization inherent to native skeletal organogenesis. However, owing to the uniquely dense, mineralized, and load-bearing nature of bone tissue, relying solely on stochastic biological self-assembly is inherently insufficient. Consequently, advanced engineering strategies—ranging from biomimetic topological scaffolds and dynamic mechanobiological stimulation to 3D bioprinting and highly integrated microfluidic systems—are not merely auxiliary tools, but indispensable morphogenetic templates that actively instruct spatiotemporal structural evolution.

Despite remarkable progress *in vitro*, bone organoids still face critical manufacturing, biological, and regulatory bottlenecks. To realize their full clinical and pharmaceutical potential, the field must move beyond isolated technical improvements. Instead, it mandates a deep convergence of multidisciplinary technologies such as biomimetic topological scaffolds, dynamic mechanobiological stimulation, 3D bioprinting, microfluidic systems, and artificial intelligence. Building upon these advanced technological foundation, successful clinical translation further requires the development of “off-the-shelf” commercial manufacturing, alongside the establishment of standardized and ethically robust evaluation frameworks. Through these comprehensive efforts, engineered bone organoids will successfully transition from laboratory models to valid alternatives for animal testing and advanced therapeutic implants. Ultimately, this multidisciplinary synergy will bridge the gap between microscopic developmental biology and macroscopic skeletal repair, reshaping the future of clinical orthopedics and personalized medicine.

## CRediT authorship contribution statement

**Jiamian Han:** Writing – review & editing, Writing – original draft, Conceptualization. **Hongcheng Gu:** Writing – review & editing, Supervision, Funding acquisition. **Zhongze Gu:** Supervision, Funding acquisition.

## Ethics approval and consent to participate

This article is a review and does not contain any studies with human participants or animals performed by any of the authors.

## Declaration of competing interest

The authors declare no conflict of interests.
